# Ca^2+^-Currents in Human Induced Pluripotent Stem Cell-Derived Cardiomyocytes Effects of Two Different Culture Conditions

**DOI:** 10.3389/fphar.2016.00300

**Published:** 2016-09-12

**Authors:** Ahmet U. Uzun, Ingra Mannhardt, Kaja Breckwoldt, András Horváth, Silke S. Johannsen, Arne Hansen, Thomas Eschenhagen, Torsten Christ

**Affiliations:** ^1^Department of Experimental Pharmacology and Toxicology, University Medical Center Hamburg-EppendorfHamburg, Germany; ^2^Partner Site Hamburg/Kiel/Lübeck, German Centre for Cardiovascular Research (DZHK)Hamburg, Germany; ^3^Department of Pharmacology and Pharmacotherapy, Faculty of Medicine, University of SzegedSzeged, Hungary; ^4^Department of General and Interventional Cardiology, University Heart Center HamburgHamburg, Germany

**Keywords:** human induced pluripotent stem cell-derived cardiomyocytes, L-type Ca^2+^-channel, T-type Ca^2+^-channel, β-adrenoceptor, 5-hydroxytryptamine, protein kinase A

## Abstract

Human induced pluripotent stem cell-derived cardiomyocytes (hiPSC-CM) provide a unique opportunity to study human heart physiology and pharmacology and repair injured hearts. The suitability of hiPSC-CM critically depends on how closely they share physiological properties of human adult cardiomyocytes (CM). Here we investigated whether a 3D engineered heart tissue (EHT) culture format favors maturation and addressed the L-type Ca^2+^-current (I_Ca,L_) as a readout. The results were compared with hiPSC-CM cultured in conventional monolayer (ML) and to our previous data from human adult atrial and ventricular CM obtained when identical patch-clamp protocols were used. HiPSC-CM were two- to three-fold smaller than adult CM, independently of culture format [capacitance ML 45 ± 1 pF (*n* = 289), EHT 45 ± 1 pF (*n* = 460), atrial CM 87 ± 3 pF (*n* = 196), ventricular CM 126 ± 8 pF (*n* = 50)]. Only 88% of ML cells showed I_Ca_, but all EHT. Basal I_Ca_ density was 10 ± 1 pA/pF (*n* = 207) for ML and 12 ± 1 pA/pF (*n* = 361) for EHT and was larger than in adult CM [7 ± 1 pA/pF (*p* < 0.05, *n* = 196) for atrial CM and 6 ± 1 pA/pF (*p* < 0.05, *n* = 47) for ventricular CM]. However, ML and EHT showed robust T-type Ca^2+^-currents (I_Ca,T_). While (−)-Bay K 8644, that activates I_Ca,L_ directly, increased I_Ca,L_to the same extent in ML and EHT, β_1_- and β_2_-adrenoceptor effects were marginal in ML, but of same size as (−)-Bay K 8644 in EHT. The opposite was true for serotonin receptors. Sensitivity to β_1_ and β_2_-adrenoceptor stimulation was the same in EHT as in adult CM (−logEC_50_: 5.9 and 6.1 for norepinephrine (NE) and epinephrine (Epi), respectively), but very low concentrations of Rp-8-Br-cAMPS were sufficient to suppress effects (−logEC_50_: 5.3 and 5.3 respectively for NE and Epi). Taken together, hiPSC-CM express I_Ca,L_ at the same density as human adult CM, but, in contrast, possess robust I_Ca,T_. Increased effects of catecholamines in EHT suggest more efficient maturation.

## Introduction

The L-type Ca^2+^-current (I_Ca,L_) is central for cardiac electrophysiology. It contributes to the shape of the cardiac action potential and its regulation plays an important role in cardiac excitability and contractility (Tsien, 1983). L-type Ca^2+^-currents are activated upon depolarization while their activity can be increased by catecholamines (Hofmann et al., 2014). Since effects of norepinephrine on action potential precede the effects on tension it is assumed that stimulation of I_Ca,L_ is related to inotropy (Reuter, 1974). Therefore, I_Ca,L_ is expected to be crucial for adapting heart function to actual needs. T-type Ca^2+^-currents are typically found in pacemaking heart cells, but are absent from the working myocardium of many adult mammalians including man (Beuckelmann et al., 1991). HiPSC-CM provide a unique opportunity to study human heart electrophysiology *in vitro* and are believed to offer a model for pharmacological drug testing as well as disease modeling (Dick et al., 2010; Hoekstra et al., 2012; Navarrete et al., 2013). Yet, hiPSC-CM display an immature cardiac phenotype, and current efforts are directed toward means to unfold the full potential of these cells by increasing their maturity (Yang et al., 2014). One such strategy could be culture in engineered heart tissue (EHT) under conditions in which hiPSC-CM form a 3-dimensional network and perform auxotonic contractile work against elastic silicone posts (Schaaf et al., 2011). Here we directly compared the biophysics and regulation of Ca^2+^-currents in hiPSC-CM cultured either in standard monolayer format (ML) or as EHT and compared the data to our previous data on human adult CM obtained under identical patch-clamp protocols.

## Materials and methods

### Differentiation of hiPSC-CM and EHT generation

Undifferentiated hiPSC (kind gift from Alessandra Moretti, Munich, Germany) were expanded in a medium which contains b**F**GF, **T**GFß1, **D**orsomorphin and **A**ctivin A [so called “FTDA” (Frank et al., 2012)], and differentiated in a three step protocol based on growth factors and a small molecule Wnt inhibitor DS07 (kind gift from Dennis Schade, Dortmund, Germany). In brief, confluent undifferentiated cells were dissociated (0.5 mM EDTA; 10 min) and cultivated in spinner flasks (30^*^10^6^ cells/100 ml; 40 rpm) for embryoid body formation overnight (Zweigerdt et al., 2011). Mesodermal differentiation was initiated in embryoid bodies over 3 days in suspension culture with growth factors (BMP-4, activing-A, FGF2). Cardiac differentiation was performed either in adhesion or in suspension culture with the Wnt-inhibitor DS07 (Lanier et al., 2012). Cells were cultured in a humidified temperature and gas-controlled incubator (37°C, 5% CO_2_, 5% O_2_; 21% O_2_ for final cardiac differentiation). At day 14 the spontaneous beating hiPSC-CM were dissociated with collagenase type II (Worthington, LS004176; 200 U/ml, 3.5 h) and either cultured in ML or EHT format. For 3-dimensional culture EHT were generated as previously described (Schaaf et al., 2014). EHT as well as ML were cultured in a 37°C, 7% CO_2_, 21% O_2_ humidified cell culture incubator with a medium consisting of DMEM (Biochrom; F0415), 10% heat-inactivated horse serum (Gibco 26050), 1% penicillin/streptomycin (Gibco 15140), insulin (10 μg/ml; Sigma I9278) and aprotinin (33 μg/ml; Sigma A1153). For further comparability, experiments were performed in parallel from the same batch of cells. After culturing hiPSC-CM in ML and EHT for 28 days cells were isolated with collagenase type II (Worthington, LS004176; 200 U/ml) for 3 h (ML) and 5 h (EHT). In order to support dissociation, trituration was performed after 1.5 and 3 h, respectively. Cells were plated on gelatin-coated (0.1%) glass coverslips for 24–48 h before patch clamp experiments were performed.

### Human adult atrial and ventricular CM

**Figure 1:**
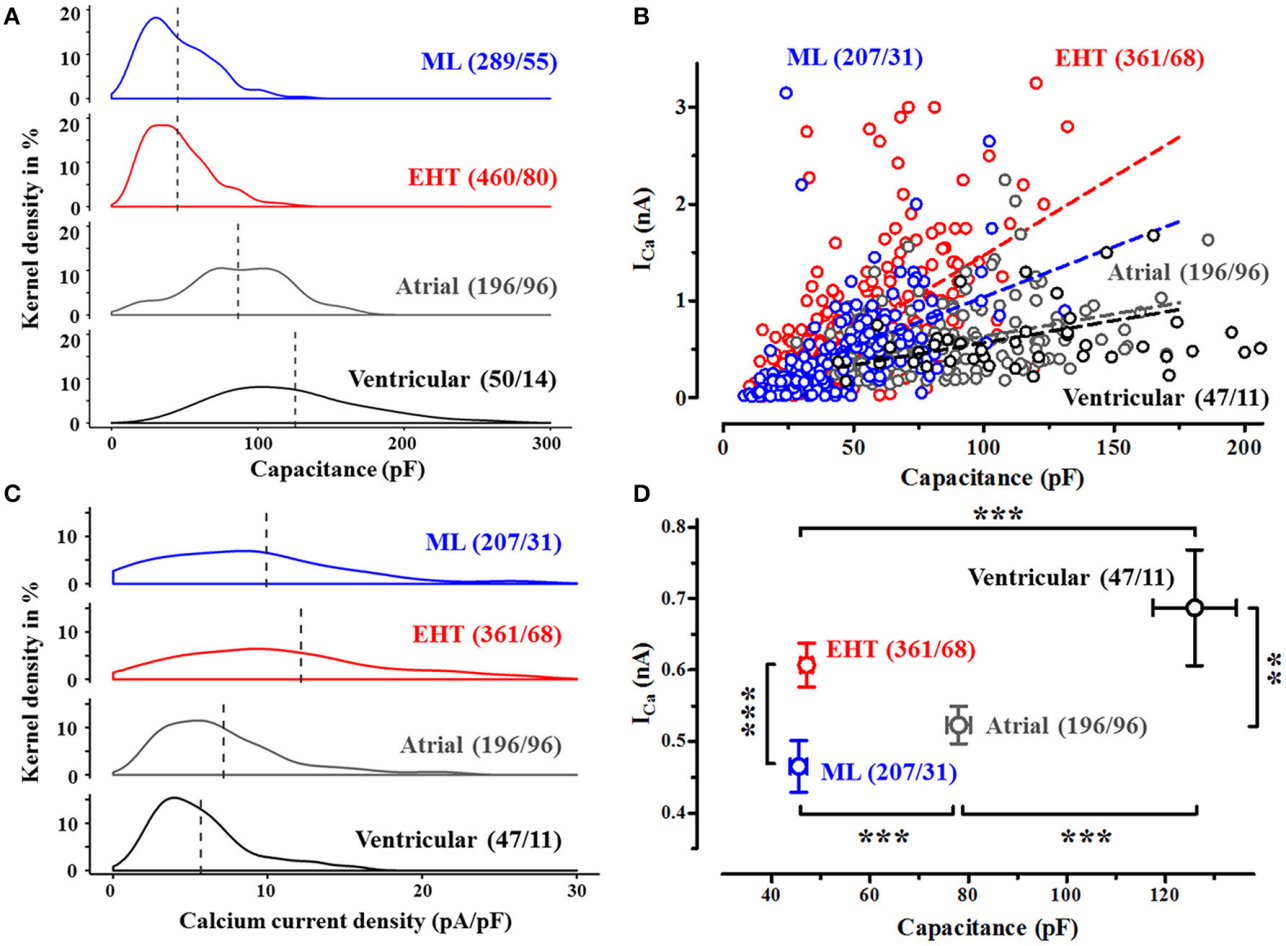
**Cell size and Ca^**2+**^-currents in human CM. (A)** Frequency distribution of cell capacitance in CM from conventional monolayer culture (ML), EHT and from human adult atrial and ventricular CM expressed as kernel density (details see methods). Dotted lines indicate mean values (compare Table S2). **(B)** Ca^2+^-current amplitudes (measured at +10 mV) vs. cell capacitance, dotted lines indicate linear regression fit (compare Table S3). Cells without ICa were not plotted. Same n-numbers as in 1C. **(C)** Frequency distribution of Ca^2+^-currents in human CM. Ca^2+^-currents are expressed as current densities, measured 3.5 min after membrane rupture (test- pulse potential +10 mV, for detailed analysis compare Table S4). **(D)** Mean values for absolute peak Ca^2+^-currents vs. cell capacitance. Please note that error bars may be smaller than symbols. Same n-numbers as in 1C. N/N indicates number of cells vs. number of isolation for ML and EHT and number of cells/number patients for atrial and ventricular CM.

**Figure 2:**
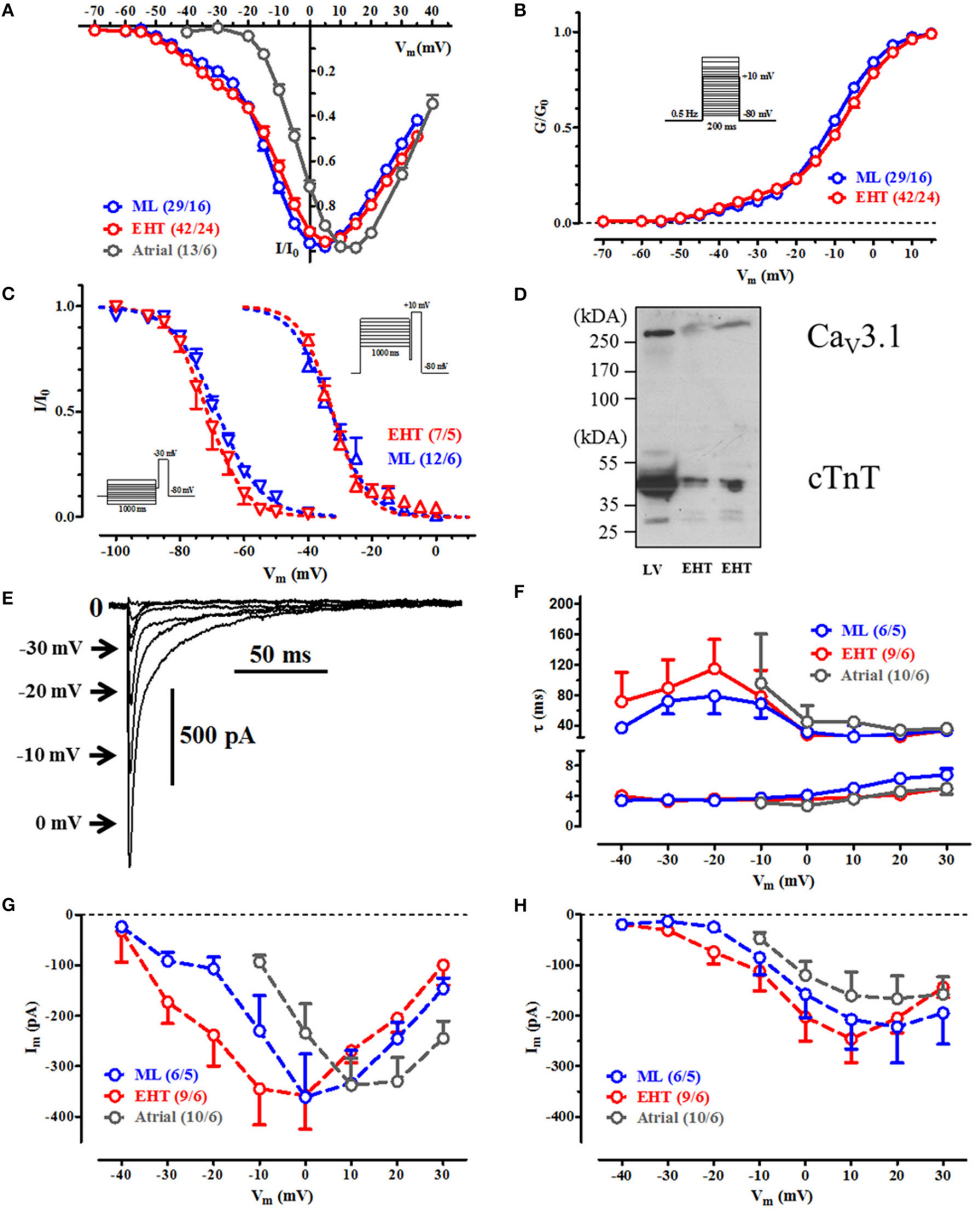
**Voltage dependency of current activation, steady state inactivation and inactivaton during a test-pulse. (A)** IV-curves for ML, EHT, and atrial CM. Currents are normalized to its individual maximum. **(B)** Activation curves for ICa. Conductances were calculated from individual IV-curves. **(C)** Steady state inactivation curves for T-type Ca^2+^-channels (at −30 mV, left) and for L-type (at +10 mV, right). **(D)** Western blot with antibodies against CaV 3.1 and cardiac troponin T (cTnT; loading control) in human adult left ventricle (LV) and engineered heart tissue (EHT). **(E–H)** Time-dependent inactivation of Ca^2+^-currents. **(E)** Original tracings of ICa during initial inactivation phase at different test pulse potentials in hiPSC-CM (For pulse protocol, compare inset in **(B)**. Numbers next to the arrows indicate respective test pulse potential, for clarity some steps were omitted). **(F)** τ-values for the fast and slow inactivation phase and respective current amplitudes obtained by curve fitting at different test-pulse potentials A **(G)** and B **(H)**. Please note that in human atrial CM no Ca^2+^-currents could be recorded at Vm < −10 mV. For details see Table S5. N/N indicates number of cells vs. number of isolation for ML and EHT and number of cells/number patients for atrial CM.

Adult myocardial tissue was obtained with informed consent from patients undergoing cardiac surgery at the Department of Heart Surgery, Dresden University of Technology. These studies were approved by the Medical Faculty Ethics Committee of Dresden University of Technology (document EK790799). Experiments were performed at the Department of Experimental Pharmacology and Toxicology, Medical Faculty, Dresden University of Technology between 2008 and 2011. Atrial and ventricular CM were isolated and prepared as previously described (Dobrev et al., 2000). Data about atrial CM displayed in Figures 1, **5D** (experiments with NE and Epi) were obtained from raw data, used for publication recently (Christ et al., 2014). Data presented in Figures 2, **4**, **5D** (only experiments with Bay K) are from new experiments done in atrial cells from 6 patients. Patients were in stable sinus rhythm and 61.7 ± 2.7 years old. The majority of patients were treated with acetylsalicylic acid, ACE-inhibitors and β-blockers (for details see Table S1).

### Western blot

For protein extraction 28-day-old EHT were frozen in liquid nitrogen and stored at −80°C. Each EHT was subjected to lysis with 70 μl 1x M-PERTM Mammalian Protein Extraction Reagent (Thermo Scientific) including protease and phosphatase inhibitor (Roche). The tissues were homogenized and supplemented with 1x Laemmli buffer prior to heating (95°C, 5 min). SDS polyacrylamide gel (8%) was loaded with 3 μl non-failing human heart tissue lysate or 10–20 μl of EHT lysate. After electrophoresis proteins were blotted onto nitrocellulose membrane using the wet blot technique. Membranes were cut, washed with TBS-Tween 0.1%, blocked in 10% in low fat milk powder solution (1–2 h) and immediately transferred for incubation with the primary polyclonal rabbit antibodies against cardiac troponin T (1:1000; Abcam, 45932) and T-type calcium channel (1:100; alomone labs, SCC-021). After washing (TBS-Tween 0.1%, 3 × 10 min) membranes were transferred to anti-rabbit IgG peroxidase-conjugated secondary antibody (1:5000, Sigma, A0545; 1 h, room temperature in 1% low fat milk powder solution). Membranes were washed with TBS-Tween (0.1%, 3 × 15 min and 0.05% 2 × 15 min) and Pierce® ECL Western Blotting Substrate (Thermo Scientific) used for band visualization.

### Whole-cell recording of I_Ca_

I_Ca_ was measured at 37°C using the whole-cell configuration of the patch-clamp technique (Axopatch 200B, Axon Instruments, Foster City, CA, USA), ISO 2 software was used for data acquisition and analysis (MFK, Niedernhausen, Germany). Heat-polished pipettes were pulled from borosilicate filamented glass (Hilgenberg, Malsfeld, Germany). Tip resistances were 2.5–5 MΩ, seal resistances were 3–6 GΩ. Cell capacitance (C_m_) was calculated from steady-state current during depolarizing ramp pulses (1V/1s) from −40 to −35 mV. Ca^2+^-currents were elicited by applying test- pulses from −80 to + 10 mV (0.5 Hz). Extracellular Ca^2+^-concentration was 2 mM. The cells were investigated in a small perfusion chamber placed on the stage of an inverse microscope. Drugs were applied with a system for rapid solution changes (Cell Micro Controls, Virginia Beach, VA, USA; ALA Scientific Instruments, Long Island, NY, USA; Christ et al., 2004a). In order to avoid contaminating currents, K^+^-currents were blocked by replacing K^+^ with Cs^+^ and tetraethylammonium-chloride in the bath solution. The experiments were performed with the following Na^+^-free bath solution (in mM): Tetraethylammonium-chloride 120, CsCl 10, HEPES 10, CaCl_2_ 2, MgCl_2_ 1 and glucose 20 (pH 7.4, adjusted with CsOH). The pipette solution (pH 7.2, adjusted with CsOH) included (in mM): Cesium methanesulfonate 90, CsCl 20, HEPES 10, Mg-ATP 4, Tris-GTP 0.4, EGTA 10, and CaCl_2_ 3 (Christ et al., 2001). For some experiments (**Figures 4A,B**) we used the technique of perforated-patch. Amphotericin B was added to the pipette solution in a concentration of 1 μM. Current amplitude was determined as the difference between peak inward current and current at the end of the depolarizing step. Steady-state inactivation curves for I_Ca_ were obtained by plotting the normalized current amplitude at the test potential as a function of the conditioning potential (V_m_). A Boltzmann function was fitted to the normalized values: I/Imax = 1/(1 + exp((V_m_ − V_0.5inact_)/k_inact_)), where V_0.5inact_ and k_inact_ are the voltage of half-maximum inactivation and the slope factor, respectively. Activation curves were calculated from current-voltage relations (IV-curves) using the equation G = I/(V_m_ − E_rev_), where G and I are peak Ca^2+^-conductance and current at the test potential V_m_, respectively. The apparent reversal potential E_rev_ was obtained by linear regression of four data points close to E_rev_. The relation between normalized peak conductance G/G_max_ and membrane potential V_m_ could be described by a bi-exponential Boltzmann equation: Y = A/(1 + exp((V_0.5actA_ − V_m_)/k_actA_)) + B/(1 + exp((V_0.5actB_ − V_m_)/k_actB_)), where V_0.5act_ is the voltage at half-maximum activation and k_act_ is the slope factor.

### Drugs

All drugs and chemicals were obtained from Sigma (St. Louis, Missouri, USA), except for Rp-8-Br-cAMPS, which was obtained from Calbiochem (Merck, Darmstadt, Germany) and (−)-Bay K 8644 from Tocris (Tocris Bioscience, Bristol, United Kingdom).

### Statistics

Results are presented as mean ± SEM. Curve fitting was performed by using the GraphPad Prism Software Version 5.02 (GraphPad Software, San Diego, CA, USA). Statistical differences were evaluated by using the Student's *t*-test (paired or unpaired) or repeated measures ANOVA, where appropriate. A value of *p* < 0.05 was considered to be statistically significant. Analyses of frequency distribution (Figures 1A,C) were performed using R (ver. 3.1.1) (R Core Team, 2013). Please note that the statistical term “Kernel density estimation” is used in Figure 1. In the first place, a “kernel” is defined as a probability density function which must possess particular properties. These are that it must be even, non-negative, real-valued and its definite integral over its support set must equal to 1. So “kernel density estimation” is a method which is non-parametric and enables to estimate the probability density function of a random variable (http://scikit-learn.org/stable/modules/density.html).

## Results

### Cell size and current density

HiPSC-CM were consistently reported to be smaller than adult CM. We could confirm this finding under our cell culture conditions. When we plotted individual cell capacitance as an electrophysiological correlate of cell size in hiPSC-CM and human adult CM, we found substantial overlap between hiPSC-CM and adult CM, but mean values in hiPSC-CM were two- to three-fold smaller than human atrial and ventricular CM, respectively (Figure 1). Cells from EHT were not larger than from ML. While all cells from EHT and also every adult CM we measured possessed robust Ca^2+^-currents (at +10 mV), a remarkable number of cells in ML (35 out of 289) did not show I_Ca_. In adult CM I_Ca_ depends on cell capacitance. Therefore, Ca^2+^-currents were normalized to cell capacitance and expressed as “current density” in order to minimize cell-to-cell variability of I_Ca_. Mean Ca^2+^-current density amounted to 9.9, 12.2, 7.1, and 5.7 pA/pF in ML, EHT, atrial and ventricular CM, respectively (Figure 1C). Despite the fact that human adult CM were clearly larger than hiPSC-CM, both human adult and hiPSC-CM showed a positive correlation between cell size and I_Ca_. However, the regression curves for I_Ca_ vs. cell capacitance were two times steeper in hiPSC-CM than in adult CM (Figure 1B). Surprisingly *R*^2^ values were unexpectedly low in all 4 populations investigated, questioning normalization of currents to cell size. In order to minimize Ca^2+^-current data scattering because of different cell size within the groups and to facilitate comparison to literature data, we will present current data as current density. As hiPSC-CM are expected to undergo maturation during culture, we have tested whether different populations of Ca^2+^-currents may exist and analyzed frequency distribution of current density in hiPSC-CM vs. adult CM. However, as in human adult CM, I_Ca_ revealed a monophasic distribution in hiPSC-CM, indicating a homogenous population.

### Voltage-dependency of activation, steady state inactivation and inactivation kinetics of I_Ca_

#### hiPSC-CM possess both low-voltage and high-voltage activated I_Ca_ (Figures 2A–C)

Next we measured voltage-dependent activation of I_Ca_ in hiPSC-CM and adult CM. In human adult CM, Ca^2+^-currents activated at test pulse potentials above −30 mV. In contrast, hiPSC-CM showed robust Ca^2+^-currents at much more negative test pulse potentials (starting already at −50 mV) giving a typical “shoulder” (Nemtsas et al., 2010). To get exact values for voltage-dependency we constructed activation curves and fitted a bi-exponential Boltzmann function to the data points. About 11–13% of total Ca^2+^-conductivity consisted of T-type Ca^2+^-current (I_Ca,T_) with activation voltage not different between ML and EHT. Steady state inactivation curves in hiPSC-CM also differed from adult CM (Mewes and Ravens, 1994; Christ et al., 2004b). Low-voltage activated I_Ca_ inactivated at more negative potentials. The data are compatible with the expression of functional T-type Ca^2+^-channels in hiPSC-CM. Accordingly, western blots with an antibody against Ca_V_3.1 showed single bands at ~270 kDa in both hiPSC-CM and a non-failing human heart sample, providing evidence for the presence of T-type Ca^2+^ channel α-subunits in hiPSC-CM (Figure 2D).

#### Time-dependent inactivation of I_Ca_ (Figures 2E–H)

Total Ca^2+^-influx critically depends not only on voltage-dependency of activation and resulting peak current amplitude but also on time-dependent inactivation at different membrane potentials. Therefore, we fitted Ca^2+^-current decay. As shown previously in human adult CM (Christ et al., 2004b), Ca^2+^-current inactivation could be fitted at positive potentials with two time constants: τ**_fast_** of ~4 ms and a τ**_slow_** of ~50 ms. Like in human adult CM, the quickly inactivating component of I_Ca_ was larger than the slowly inactivating component. In summary we found the same biophysical properties of L-type Ca^2+^-currents as in adult CM.

### Pharmacological block of I_Ca_

#### Nifedipine does not completely block I_Ca_ in hiPSC-CM (Figure 3)

**Figure 3:**
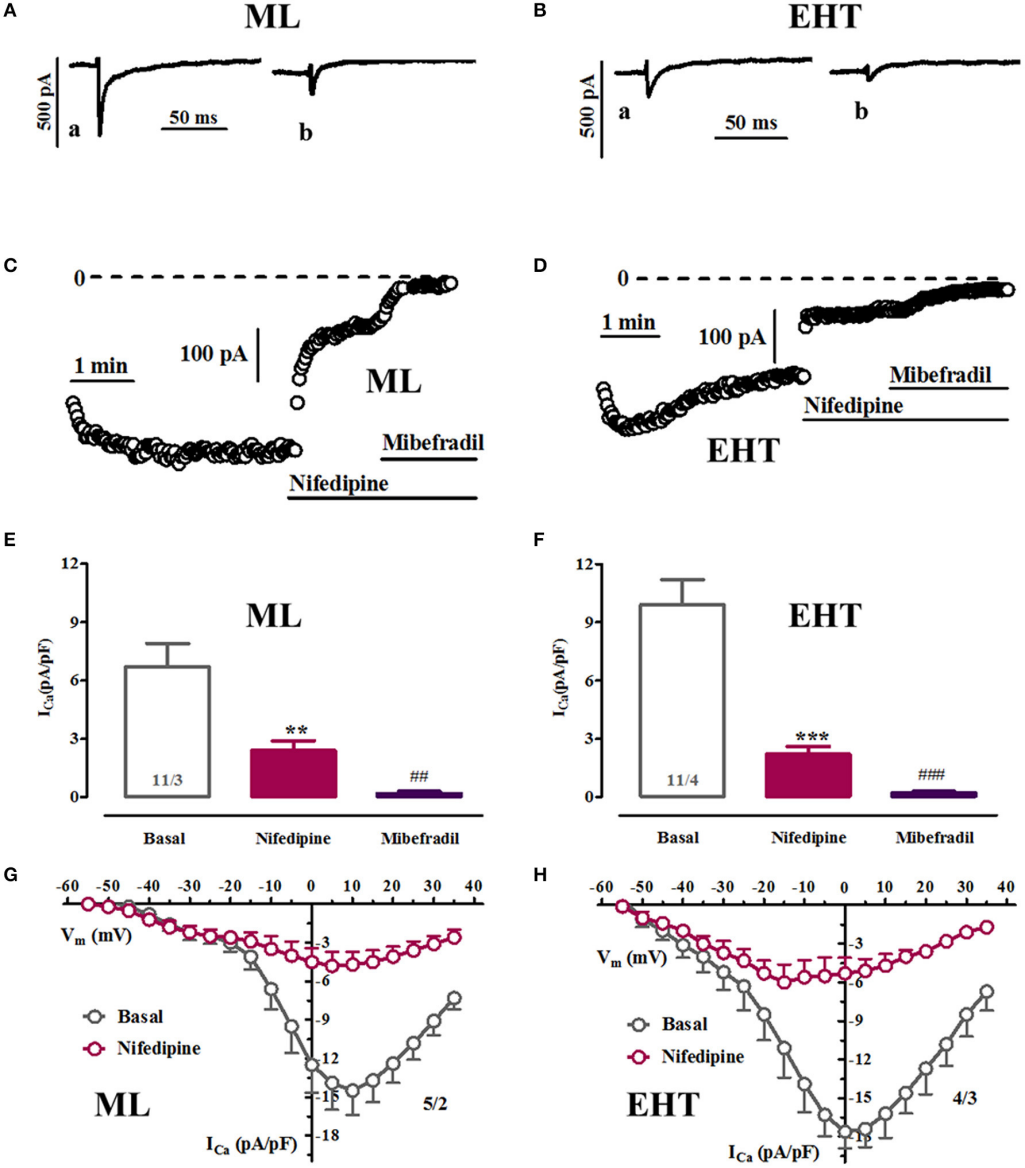
**Pharmacological discrimination of Ca^**2+**^-currents in ML and EHT. ML and EHT were exposed to nifedipine and mibefradil. (A–D)** Respective time courses taken in ML **(A,C)** or EHT **(B,D)** from individual experiments. Original tracings taken at time points indicated as a and b. **(E,F)**: Ca^2+^-currents measured at +10 mV in ML **(E)** and EHT **(F)** exposed to nifedipine (10 μM) and on top to mibefradil (10 μM). ^**^p < 0.05 and ^***^p < 0.001 vs. basal and ^##^*p* < 0.01 and ^###^*p* < 0.001 vs. nifedipine. **(G,H)** IV-curves recorded under control condition and in the presence of 10 μM nifedipine in ML **(G)** and EHT **(H)**. N/N indicates number of cells vs. number of isolation for ML and EHT.

To further evaluate the hypothesis that I_Ca_ in hiPSC-CM contains I_Ca,T_ we applied pharmacological blockers. We used nifedipine to block L-type and mibefradil to block T-type Ca^2+^-currents. Since the selectivity of mibefradil is moderate compared to the selectivity of nifedipine, cells were exposed first to nifedipine and mibefradil was added on top (Figures 3E,F). Exposure to high concentrations of nifedipine (10 μM) did not block Ca^2+^-currents completely (Figure 3). Scrutinizing IV-curves in the presence of nifedipine revealed that a large amount of the remaining current measured at +10 mV should mainly result from T-type Ca^2+^-currents. In line with this assumption, mibefradil blocked the nifedipine-insensitive current at +10 mV. The data suggest that a substantial amount of Ca^2+^-currents at +10 mV relates to I_Ca,T_, both in ML and EHT. This interpretation is complicated by the so-called slip-mode conduction in which, in a Na^+^-free environment, the Na^+^-channel can also conduct Ca^2+^ ions, thereby mimicking I_Ca,T_ (Mitra and Morad, 1986; Heubach et al., 2000). Since mibefradil not only blocks I_Ca,T_ but also slip-mode conduction (Heubach et al., 2000) we employed tetrodotoxin (TTX; 30 μM). However, the Ca^2+^-currents at low test pulse potentials were insensitive to TTX, confirming the assumption of I_Ca,T_. Furthermore, I_Ca,L_ was also insensitive to TTX (Figure S1).

### Run-down and activation of I_Ca,L_ by (−)-Bay K 8644

**Figure 4:**
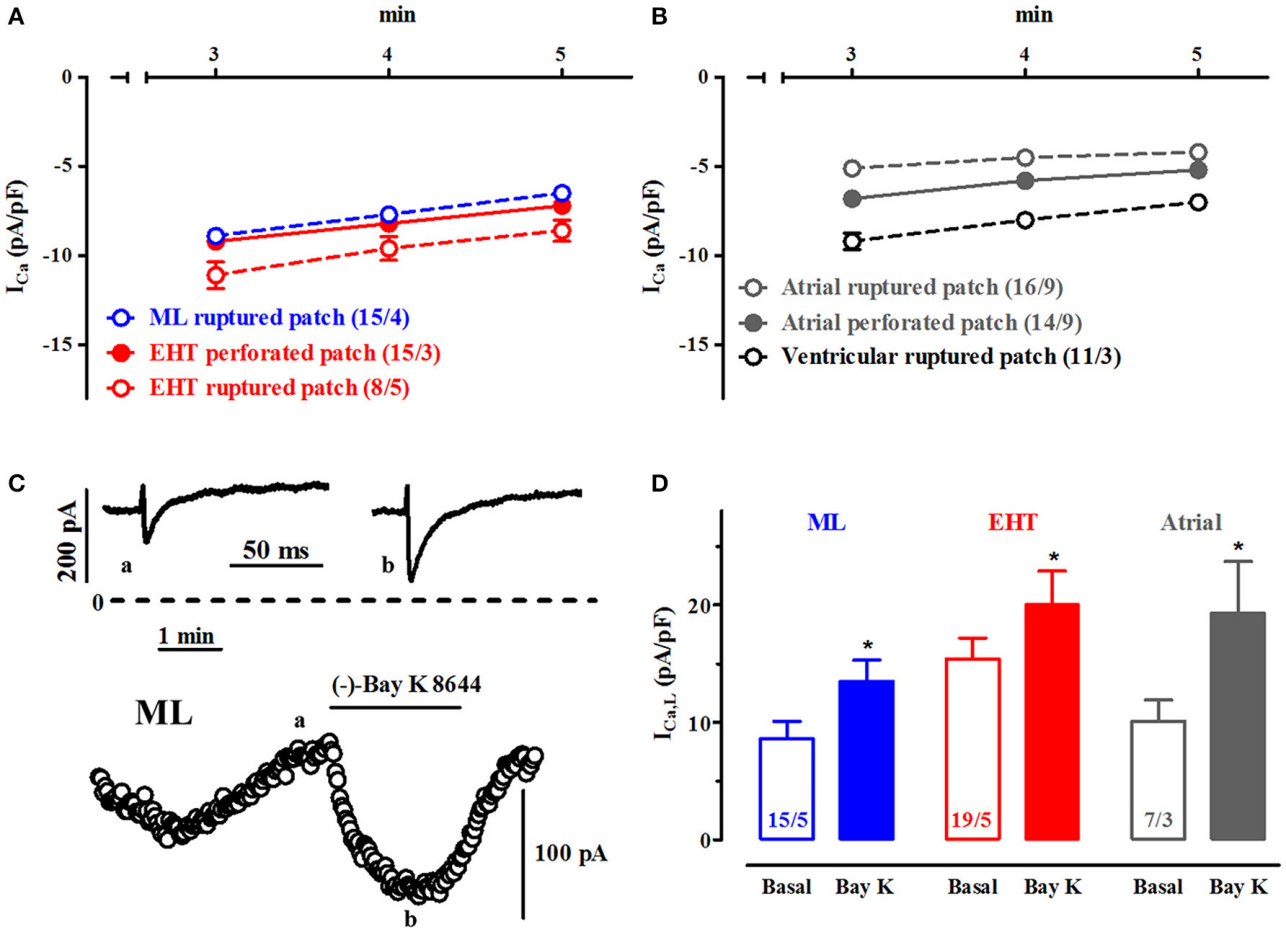
**Run-down and activation of ICa,L by (−)-Bay K 8644 in ML and EHT. (A,B)** Run-down of ICa: Mean ICa densities during the time frame of experiments in time-matched controls in ML and EHT **(A)** and in adult CM **(B)** (please note data for perforated patch given with closed symbols). **(C,D)** Effects of (−)-Bay K 8644: **(C)** Time-course and original tracings for time points a and b of ICa,L in response to 10 μM (−)-Bay K 8644 in ML. **(D)** Mean values for ICa,L before and after (−)-Bay K 8644 (10 μM) in ML, EHT and atrial CM (^*^ indicates *p* < 0.05 vs. respective basal values). N/N indicates number of cells vs. number of isolation for ML and EHT and number of cells/number patients for atrial CM.

Ca^2+^-currents in adult CM typically decrease over time, often with an initial rapid phase and stabilization over time even when the technique of perforated-patch was used. In hiPSC-CM we could not reach stable current densities in the time frame of 5 min. Since hiPSC-CM were found extraordinary fragile compared to human adult cardiomyocytes, we looked for a compromise. Therefore, we decided to measure basal current characteristics already 3.5 min after membrane rupture. In order to evaluate whether L-type Ca^2+^-currents in hiPSC-CM share the typical response to a Ca^2+^-channel opener we employed the dihydropyridine derivative (−)-Bay K 8644. The (−)-enantiomer was used as the (+)-enantiomer blocks I_Ca,L_ and may thereby impair the maximal drug response (Ravens and Schoepper, 1990; Ji et al., 2014). High concentrations of (−)-Bay K 8644 (10 μM) increased Ca^2+^-currents in ML, EHT and adult atrial CM by 57.0, 29.9, and 91.1%, respectively (Figure 4D). The effect size did not differ between ML and EHT when expressed as delta values (increase by 4.8 and 4.7 pA/pF, respectively).

### Activation of I_Ca,L_ by catecholamines

#### Both β_1_- and β_2_-AR stimulation increase currents in hiPSC-CM

**Figure 5:**
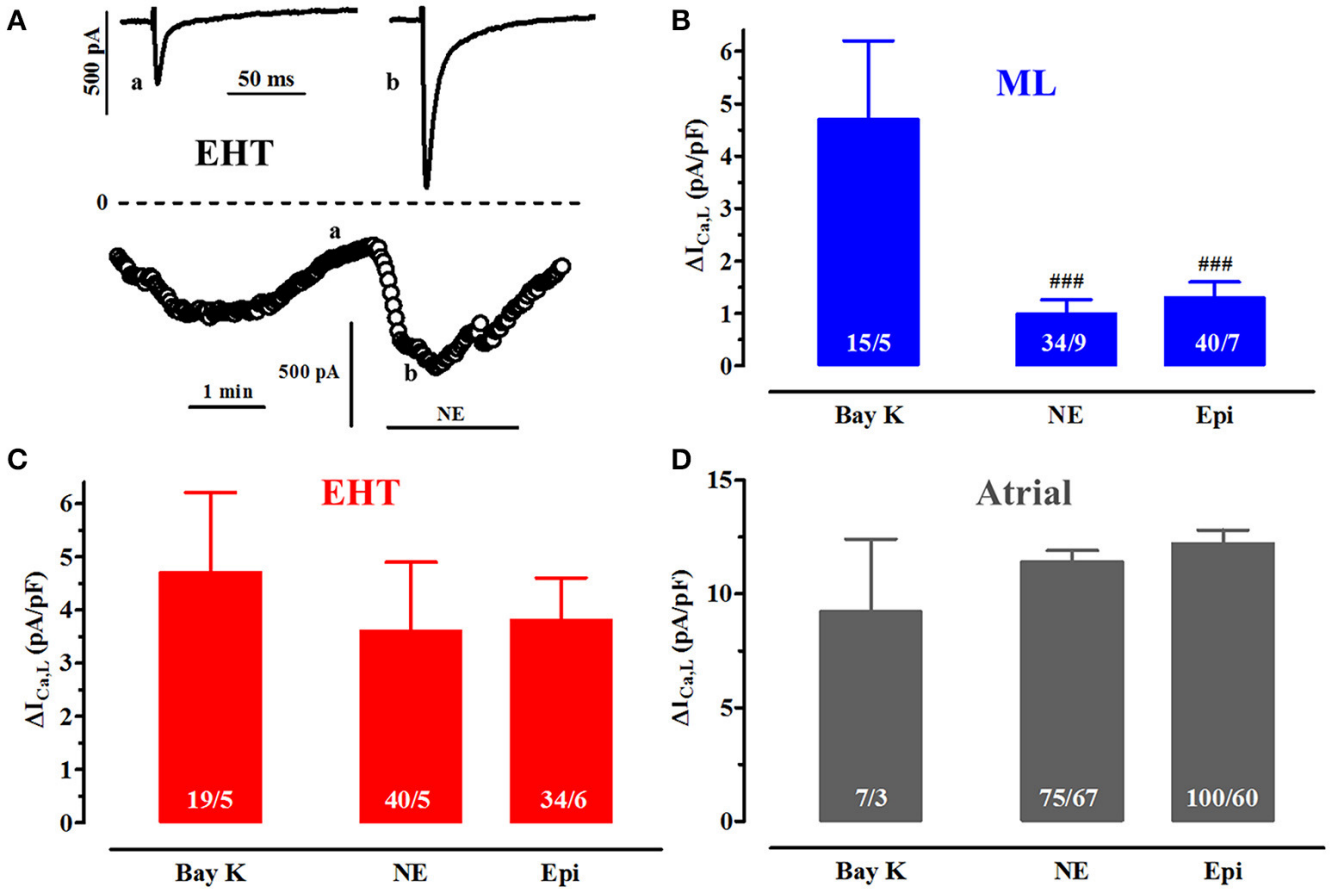
**Catecholamine effects vs. direct activation of ICa,L. (A)** Time course of ICa,L upon exposure to norepinephrine (NE, 100 μM) in a CM from EHT. Original tracings taken at time points indicated as a and b. **(B–D)** Mean delta values for the increase in ICa,L expressed as current densities upon stimulation of β1-AR with NE (100 μM), β2-AR with epinephrine (Epi, 100 μM) in the presence of the respective β-AR antagonist. Results from ML **(B)**, EHT **(C)**, and atrial CM **(D)**. Data for (−)-Bay K 8644 were taken from Figure 4 and given as delta values for comparison. # indicates significance vs. (−)-Bay K 8644. Please note different y axis scaling in D! N/N indicates number of cells vs. number of isolation for ML and EHT and number of cells/number patients for atrial CM.

In the human heart I_Ca,L_ is under regulation by β_1_- and β_2_-AR. To activate β_1_-AR we used norepinephrine (NE) in the presence of the selective β_2_-AR antagonist ICI118,551 (50 nM) and to activate β_2_-AR epinephrine (Epi) in the presence of the β_1_-AR antagonist CGP 20712A (300 nM; Kaumann et al., 1989). To assess maximum effects we used first very high concentrations of NE and Epi (100 μM each) and compared effect size to direct activation by (−)-Bay K 8644 (See Run-down and activation of I _Ca,L_ by (−)-Bay K 8644). Activation of β_1_- and β_2_-AR increased I_Ca,L_ in both ML and in EHT. The onset of the effect was as fast as in adult CM which is within ~20 s (Christ et al., 2006). The effect of β_1_ AR equaled that of β_2_ AR stimulation in ML, EHT, and adult atrial CM. However, while β_1_- and β_2_-AR effects matched that of (−)-Bay K 8644 in EHT and atrial CM (Figures 5C,D), they were much lower in ML (Figure 5B). Of note, even in EHT the absolute increases induced by catecholamines or (−)-Bay K 8644 were smaller than in adult CM. One explanation might be that L-type Ca^2+^-currents in hiPSC-CM are already high at baseline and cannot be increased any further. However, when plotted against each other, larger basal current densities were associated with larger current increases (Figure S2), arguing against this hypothesis. Steeper linear regression curves in adult CM than in both hiPSC-CM groups also indicate that the smaller I_Ca_ responses in hiPSC-CM are not related to basal properties.

### Catecholamine sensitivity

#### Sensitivity in hiPSC-CM is not different from adult CM (Figure 6)

**Figure 6:**
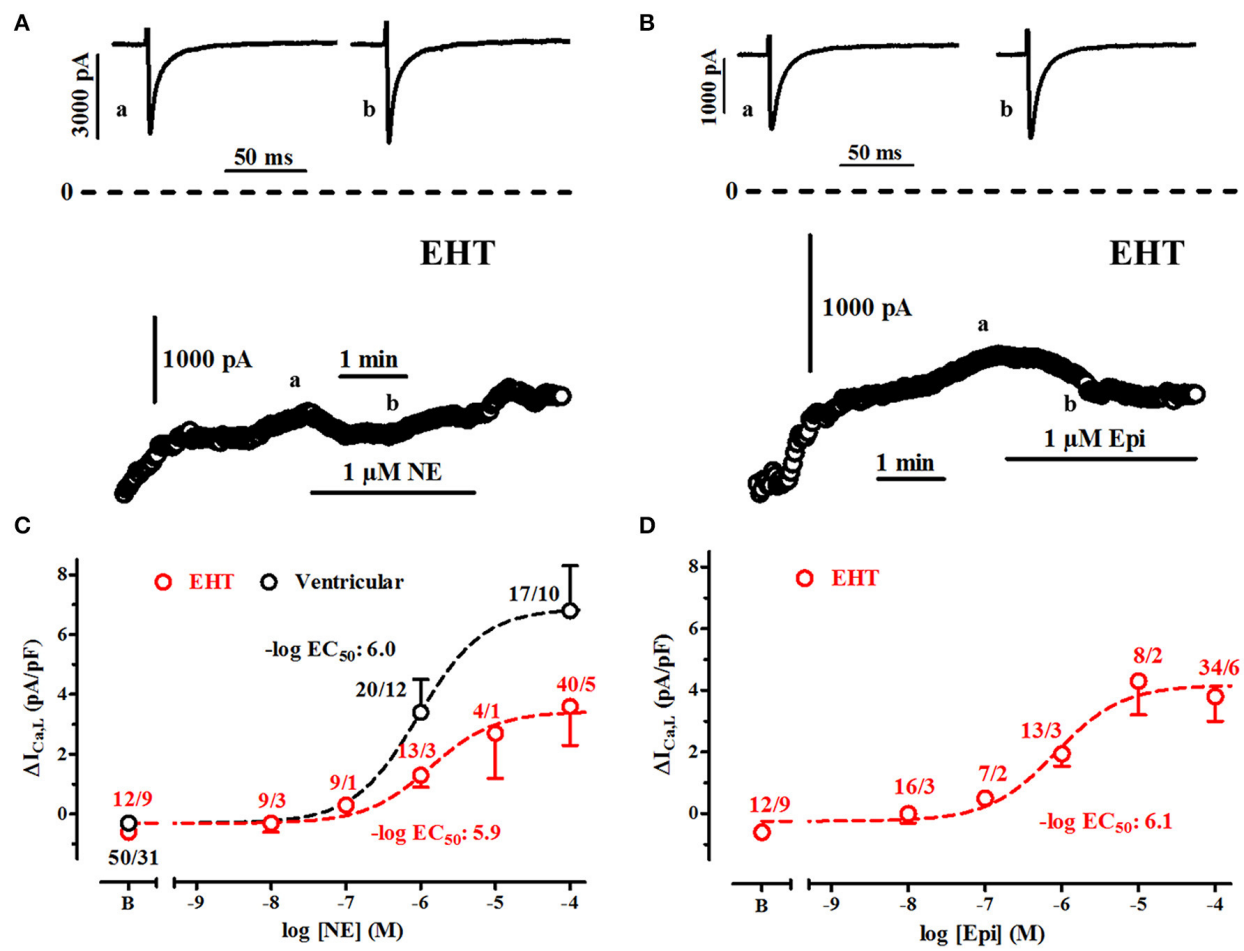
**Sensitivity of ICa,L to stimulation of β1-AR and β2-AR. (A,B)** Time course of ICa in EHT exposed to norepinephrine (NE, 1 μM) and epinephrine (Epi, 1 μM). Original tracings taken at time points indicated as a and b. **(C,D)** Concentration-response curves for the increase of ICa,L upon stimulation of β1-AR **(C)** and β2-AR **(D)**. Effects are expressed as delta values. Data from ventricular CM are given for comparison in C only. N/N indicates number of cells vs. number of isolation for EHT and number of cells/number patients for ventricular CM.

Increases in I_Ca_ by high concentrations of catecholamines were smaller in EHT than in adult CM. Either maximum effects could be diminished or sensitivity is so low that even 100 μM did not induce maximum effects. Therefore, we have measured concentration-response curves for increase of I_Ca,L_ by β_1_- and β_2_-AR stimulation in EHT. Due to small effect size we refrained from doing so in ML. To avoid complications because of run-down and desensitization of receptors catecholamines were applied in a non-cumulative manner. The threshold concentration for both NE- and Epi-induced increases in I_Ca_ was 100 nM and calculated −log EC_50_ values amounted to 5.9 and 6.1, respectively (Figures 6C,D). These values do not differ from those determined in human adult ventricular CM and adult atrial CM (Christ et al., 2014).

### CAMP-dependency of I_Ca,L_-increase due to β_1_- and β_2_-AR stimulation

#### β_1_- and β_2_-AR-mediated increases can be suppressed by much lower concentrations of Rp-8-Br-cAMPS than in adult CM (Figure 7)

**Figure 7:**
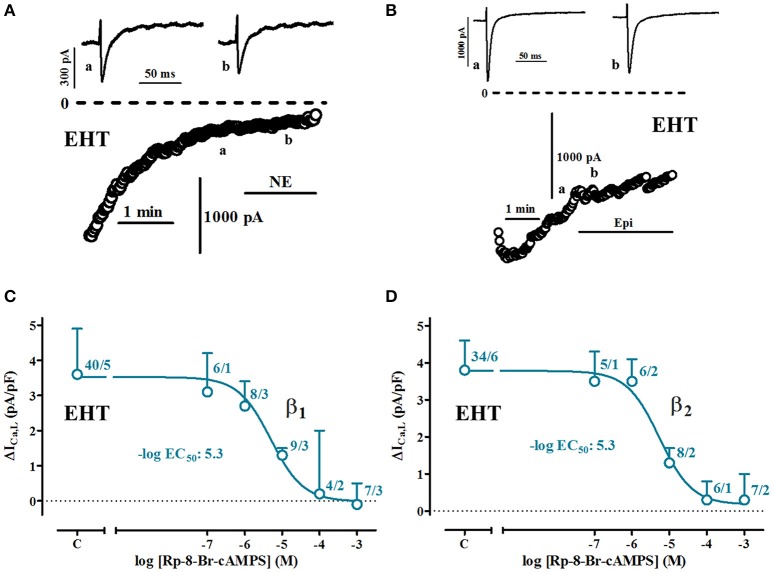
**PKA-dependency of β1-and β2-AR-mediated increases of ICa,L in EHT. (A,B)** Time course of ICa in cells exposed to norepinephrine (NE) and epinephrine (Epi, each 100 μM) when intracellularly perfused with Rp-8-Br-cAMPS (1 mM). Original tracings taken at time points indicated as a and b. **(C,D)** Concentration-response curves for the inhibition of β1-and β2-AR mediated increases of ICa,L in EHT. In all experiments concentration of NE or Epi was 100 μM. N/N indicates number of cells vs. number of isolation for EHT.

Patch-clamp experiments in isolated cells give the unique opportunity to intracellularly apply inhibitors of signal transduction. In order to estimate cAMP levels relevant for I_Ca_ activation we employed Rp-8-Br-cAMPS, a compound competitively inhibiting the effects of native cAMP on PKA (Figure 7). We measured effects of different concentrations of Rp-8-Br-cAMPS on basal currents and currents activated by maximum concentrations of catecholamines (100 μM). Since Ca^2+^-current increases by catecholamines in ML were small and hardly detectable in many experiments, we restricted the analysis to EHT. The presence of Rp-8-Br-cAMPS in the patch pipette did not decrease basal currents (data not shown). Both β_1_- and β_2_-AR mediated increases in EHT could be suppressed concentration-dependently with calculated −logEC_50_ values of 5.3 (Figures 7C,D). Compared to previous data from human adult atrial CM (Christ et al., 2014), −logEC_50_values were 20 times lower in EHT.

### 5-HT-effects on I_Ca_

#### 5-HT increases I_Ca_ in hiPSC-CM less than in atrial CM (Figure 8)

**Figure 8:**
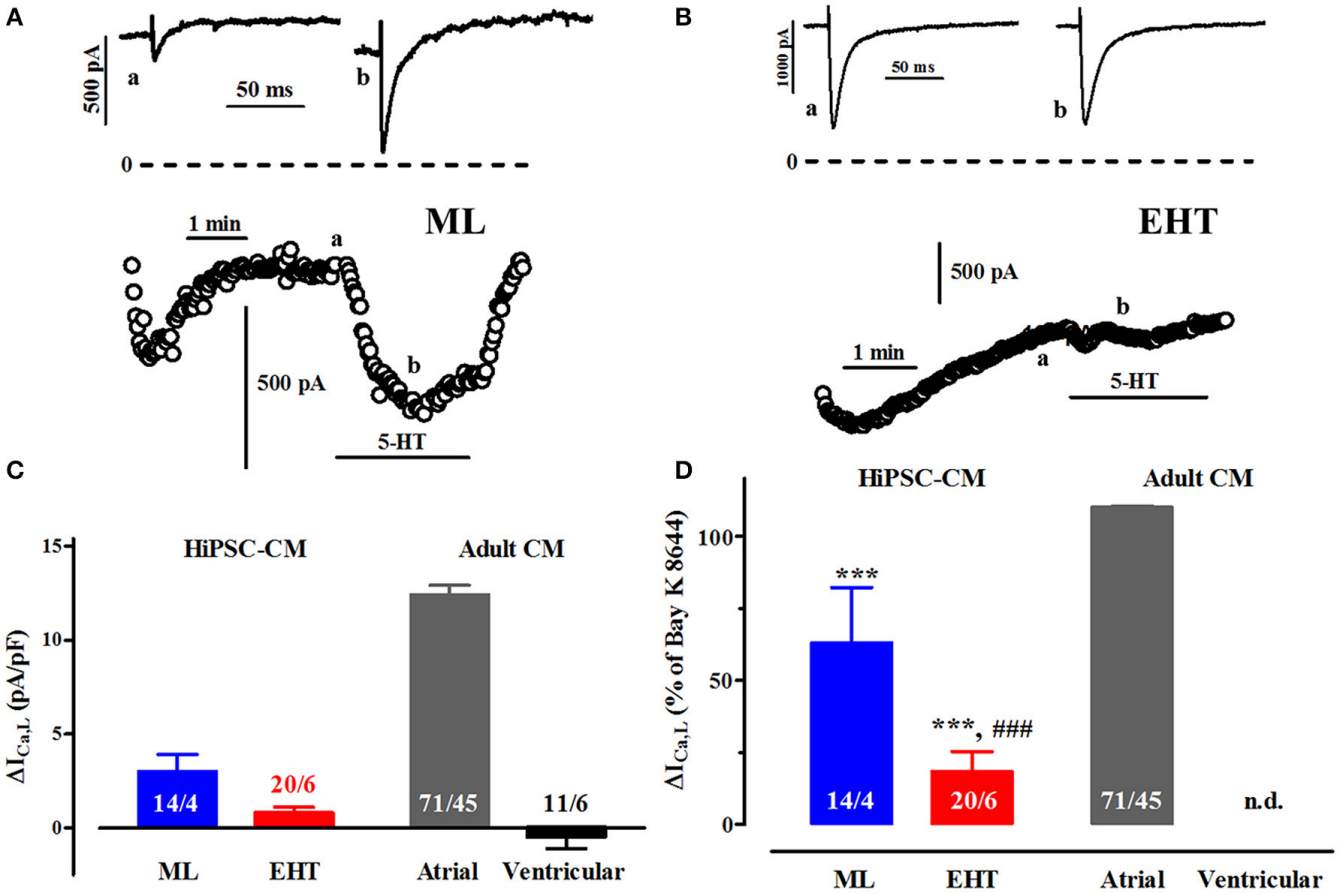
**Effects of serotonin on ICa. (A,B)** Time course of ICa upon exposure to serotonin (5-HT, 100 μM) in ML **(A)** and EHT **(B)**. Original tracings taken at time points indicated as a and b. **(C)** Mean values for the increase in ICa,L expressed as delta (Δ) current densities in ML, EHT, atrial and ventricular CM. Please note negative values for ventricular cells indicate spontaneous run-down not different from time-matched controls (data not shown). **(D)** Mean values for the 5-HT-induced increases in ICa,L in ML, EHT, and atrial CM (taken from Figure 8C) expressed as percentage of response to (−)-Bay K 8644 (calculated from Figure 5). Increase in percent not calculated for ventricular CM. ^*^ indicates significance vs. atrial and # vs. ML respectively. N/N indicates number of cells vs. number of isolation for ML and EHT and number of cells/number patients for atrial and ventricular CM.

Serotonin (5-HT) exerts positive inotropic effects in ventricular preparations from newborn, but not adult pigs (Jahnel et al., 1992; Schoemaker et al., 1992). In contrast, 5-HT-inotropy persists in atrial preparations from pigs (and even humans; Christ et al., 2014). The lack of positive inotropy in human ventricle could be related to the inability of 5-HT to increase I_Ca_, albeit evidence is based on very preliminary data (Jahnel et al., 1992). We therefore evaluated the effect of 5-HT (100 μM) on Ca^2+^-currents in hiPSC-CM, indicative of an immature and/or atrial-like phenotype (Figure 8). First we confirmed the failure of 5-HT to raise I_Ca_ in human ventricular CM in a larger number of cells. In atrial CM 5-HT-evoked increases were similar compared to direct Ca^2+^-channel activation by (−)-Bay K 8644. HiPSC-CM showed increases in I_Ca_ (Figure 8C), but at much smaller size than in atrial CM. While increases in ML cells accounted to 60% of (−)-Bay K 8644-effects, they amounted to only 20% in EHT, indicating more advanced maturation or ventricular differentiation.

## Discussion

Here we evaluated whether Ca^2+^-channels in hiPSC-CM share typical properties and regulatory mechanisms of Ca^2+^-channels in human adult CM and whether advanced culture conditions in EHT favor maturation. We found many similarities but also distinct differences between hiPSC-CM and adult human CM: (I) Basal I_Ca,L_ density was not smaller in hiPSC-CM than in adult CM. (II) HiPSC-CM, other than adult human CM, express T-type Ca^2+^-currents and the necessary pore forming α-subunit (Figure 2D). (III) Voltage-dependency of activation, steady state inactivation and inactivation kinetics of I_Ca,L_ in hiPSC-CM were not different from those found in adult CM (IV) I_Ca,L_ in hiPSC-CM was increased upon β_1_- and β_2_-AR stimulation with the same sensitivity as in adult human CM, but maximum effect size was smaller. (V) Catecholamine-induced increases of I_Ca,L_ in hiPSC-CM were PKA-dependent, but the amount of cAMP related to Ca^2+^-increase was less.

### Cell size

Smaller than normal mean cell size is considered as one of the hallmarks of hiPSC-CM (Yang et al., 2014). Mean cell size in hiPSC-CM as determined by cell capacitance in a large number of cells was approximately two-fold and three-fold smaller than atrial and ventricular CM, respectively (45.2 vs. 86.7 and 125.7 pF). While the data principally confirm previous conclusions (Yang et al., 2014), absolute values were almost three-fold higher in our hiPSC-CM (45.2 vs. 17.5 pF). The reasons are not clear, but may be related to different differentiation and culture protocols. Of note, cell capacitance/size showed substantial overlap between hiPSC-CM and adult atrial and even ventricular CM, indicating that at least some hiPSC-CM reach an adult-like size. Identical values in ML and EHT imply that the EHT-format did not have favorable effects on this parameter of CM maturity. Small size represents technical limitations in patch-clamp as discussed by Wilson et al. (2011). However, analyzing cell size and Ca^2+^-currents revealed a rather monotonic frequency distribution and Ca^2+^-currents showed linear dependency on cell size also in the low cell size range. Therefore, we believe that the relatively small cell size of hiPSC-CM is not a major obstacle for the measurement of large membrane currents such as Ca^2+^-currents.

### Ca^2+^-current density in hiPSC-CM

Few studies measured Ca^2+^-currents in hiPSC-CM and reported current densities of 3.3–17.1 pA/pF (Ma et al., 2011; Yazawa et al., 2011). Keeping in mind that results are hardly comparable due to different methodology, we compared our measurements from hiPSC-CM to previous data on human adult atrial as well as ventricular CM under identical experimental conditions. I_Ca,L_ was undetectable in 10% of cells isolated from conventional ML, indicating either a very low level of cardiac differentiation or a by-chance picking of a non-cardiac cell, present in our differentiation protocol at ~10–15%. In contrast, all cells from EHT showed robust Ca^2+^-currents. Absolute Ca^2+^-current amplitudes in hiPSC-CM did not differ significantly from adult atrial and ventricular CM, but, at smaller mean cell size, mean current density tended to be larger in hiPSC-CM (9.9–12.2 vs. 5.7–7.1 pA/pF), giving a potentially wrong impression of basal “overactivity” of Ca^2+^-currents in hiPSC-CM.

### Biophysical and pharmacological properties of I_Ca_

#### Low-voltage activated I_Ca_: I_Ca,T_ or I_Ca,TTX_?

The contribution of Ca^2+^-currents to depolarization and to transsarcolemmal Ca^2+^-influx depends on the voltage-dependency of every individual Ca^2+^-channel type expressed and their relative amplitude. Our data in hiPSC-CM showed Ca^2+^-currents activating at low and high voltage, suggesting contribution of T-type to total Ca^2+^-influx over the physiological voltage range. Several observations support this hypothesis. Besides the typical voltage for half-maximum activation between 32 and 37 mV (Hansen et al., 2004), western blots from hiPSC-CM showed a robust signal for Ca_v_3.1, the ion channel subunit carrying I_Ca,T_ (Hansen et al., 2004). The highly specific L-type blocker nifedipine reduced total I_*Ca*_ only by ~80% and the remainder was sensitive to the non-selective I_Ca_ blocker mibefradil. However, measuring Ca^2+^-currents at low voltages can raise complications since Na^+^-channels in the absence of Na^+^ can conduct Ca^2+^ to some extent (slip-mode conduction) and thereby give a wrong impression of I_Ca,T_(Mitra and Morad, 1986). Even worse, slip-mode conduction is at least in part sensitive to mibefradil (Heubach et al., 2000). In order to discriminate whether currents we measured at low voltages may represent an artifact or not, we employed tetrodotoxin (TTX; Lemaire et al., 1995). Thirty microliter of TTX is expected to block slip-mode conduction completely, but does not affect I_Ca,T_ (Mitra and Morad, 1986; Heubach et al., 2000). In our experiments Ca^2+^-currents at low voltage were completely insensitive to TTX, providing further evidence for the existence of functional T-type Ca^2+^-channels in ML and EHT (Figure S1). Unexpectedly, in contrast to many classic pharmacological studies, I_Ca,L_ was reported to be sensitive to high concentrations of TTX (>30 μM). However, results were obtained in canine cardiomyocytes only (Hegyi et al., 2012). There are no data on human cardiomyocytes.

#### I_Ca,T_ in hiPSC-CM

The finding of T-type Ca^2+^-currents in hiPSC-CM is important. Former studies characterizing Ca^2+^-currents in hiPSC-CM did either not support the existence of T-type Ca^2+^-currents (Ma et al., 2011) or did not address this question (Yazawa et al., 2011). T-type currents were found in ventricular CM from fishes (Maylie and Morad, 1995; Nemtsas et al., 2010), dogs, guinea pig and sinoatrial node cells from rabbit (Mitra and Morad, 1986; Hagiwara et al., 1988; Hirano et al., 1989). In human adult atrial and ventricular myocardium, T-type Ca^2+^-currents were consistently absent (Beuckelmann et al., 1991; Ouadid et al., 1991; Li and Nattel, 1997; Bosch et al., 1999). Data about T-type Ca^2+^-channel in development of human heart are understandably rare. Qu and Boutjdir (2001) found a decline of T-type Ca^2+^-channel mRNA expression by RT-PCR in fetal ventricular tissue over time of development. Kawano and DeHaan (1989, 1990) found large T-type Ca^2+^-channel amplitudes in chicken embryos, but no change over time. T-type Ca^2+^-currents in rat atrial CM dropped only slightly during postnatal development (Xu and Best, 1992). Therefore, it remains unclear whether I_Ca,T_ is an indicator of cardiac myocyte immaturity (Ono and Iijima, 2010). While the role of I_Ca,T_ in pacemaking is established (Marger et al., 2011; Mesirca et al., 2014), its relevance in the working myocardium is less clear. Effects of β-AR stimulation seem to be species-dependent, with stimulation in dog and guinea pig ventricular CM (Mitra and Morad, 1986; Tseng and Boyden, 1991), but no effects in shark (Maylie and Morad, 1995). In our cells the T-type Ca^2+^-current was insensitive to β-AR stimulation (Figure S3). Taken together, the co-existence of T-type and L-type Ca^2+^-channels in hiPSC-CM suggests a permanent inward current at low potentials. Such a current very likely contributes to the (abnormal) pacemaking in these cells. Further studies have to clarify the long-term functional relevance of I_Ca,T_ in hiPSC-CM.

### L-type Ca^2+^-currents in hiPSC-CM show classic pharmacological properties: (−)-Bay K 8644

In cellular electrophysiology individual membrane currents were frequently identified by selective blockers. I_Ca,L_ cannot only be blocked but also be activated, for example by (−)-Bay K 8644 (Schramm et al., 1983). Because the compound circumvents activation via intracellular signaling cascades it is often used to estimate maximum activity of L- type Ca^2+^-channels (Ouadid et al., 1991). Accordingly, Ji et al. (2014) employed the agent recently in commercially available hiPSC-CM (Cor.4U, Axiogenesis, iCell, Cellular Dynamics International) and could detect increases in currents only when Ba^2+^ was used as charge carrier (eliminating Ca^2+^-dependent inactivation of Ca^2+^-channels) and cells were hold at low potentials to increase the affinity of (−)-Bay K 8644. In contrast, (−)-Bay K 8644 robustly increased I_Ca,L_ both in ML and EHT in our hands. The discrepancy could be due to differences in the experimental protocol. For example, application of (−)-Bay K 8644 early after patch rupture may interfere with the initial fast run-down phase. More likely, however, the discrepant results with (−)-Bay K 8644 indicate a different biology of Ca^2+^-channels in cells from Cor.4U and iCell compared to our hiPSC-CM. Since we found (−)-Bay K 8644 effect sizes to be independent from culture condition (EHT vs. ML), differences in the differentiation protocol [e.g., growth factor-based (Burridge et al., 2012)] vs. small molecule-based (Burridge et al., 2014) may be more likely underlying. Head-to-head investigations are needed to clarify this issue.

### Catecholamine responses are smaller in ML than in EHT

In human (in contrast to rat and mouse) adult myocardium activation of β_1_- and β_2_-AR increases I_Ca,L_ to the same extent (Christ et al., 2014). Absolute current increase depends on temperature (Christ et al., 2011). We could confirm both findings for hiPSC-CM (Figure S4). Culture conditions had a main impact on catecholamine responses. While catecholamine effects equaled that of (−)-Bay K 8644 in EHT, effects in ML were clearly smaller. Both hiPSC-CM responses were smaller than in adult atrial or ventricular CM. It should be noted, that effect size is smaller in ventricles compared to atria. The differences are not due to higher baseline current density, because current density was positively associated with the β-AR response in all preparations. The sensitivity for activation of β_1_-AR by NE and β_2_-AR by Epi was identical to adult ventricular CM (this study) and adult atrial CM (Christ et al., 2014). Smaller maximum effect size but preserved sensitivity could indicate proper AR function but reduced ability of adenylate cyclase to generate cAMP. In order to estimate the amount of cAMP activating I_Ca,L_, we measured maximum effects in the presence of different concentrations of Rp-8-Br-cAMPS, which inhibits binding of cAMP to PKA competitively (Van Haastert et al., 1984). As shown before for human adult CM (Christ et al., 2014), basal current activity was independent from PKA activity, but catecholamine effects were concentration-dependently suppressed. The observation that 20 times less Rp-8-Br-cAMPS was sufficient to inhibit catecholamine-induced effects in EHT than in adult atrial CM (Christ et al., 2014), indicates that the small β-AR-effect on I_Ca,L_ in EHT could be related to lower β-AR-induced cAMP-generation by an immature β-AR/Gs-protein/adenylyl cyclase signaling complex.

### 5-HT increases I_Ca,L_ in hiPSC-CM: indicator for immaturity or just atrial-like phenotype?

Expression of 5-HT-receptor transcripts (RT-PCR) is higher during fetal development, and increased expression of 5-HT-receptors in adult myocardium under pathological conditions is interpreted as fetal (Brattelid et al., 2012). Data for I_Ca,L_-reponses to 5-HT from fetal heart cells are lacking. In human adult heart 5-HT-responses are restricted to atrium (Kaumann et al., 1990; Ouadid et al., 1992). 5-HT effects on I_Ca,L_ in hiPSC-CM indicate an atrial and/or immature phenotype. Smaller 5-HT-effects together with larger catecholamine effects indicate that EHT favor functional maturation compared to standard ML.

## Conclusions

In hiPSC-CM from both ML and EHT we found I_Ca,L_ not smaller than in human adult CM. Basal current densities as well as current increases to (−)-Bay K 8644 did not differ between ML and EHT. However, only hiPSC-CM from EHT showed robust catecholamine responses, suggesting maturation of the β-AR/Gs-protein/adenylyl cyclase signaling complex.

## Limitations

We used ventricular CM obtained from patients with end-stage heart-failure only. I_Ca,L_ responses are reported to be reduced in heart failure by Chen et al. (2002), whereas Mewes et al. (Mewes and Ravens, 1994) demonstrated unchanged responses. In both studies basal currents were not affected (Mewes and Ravens, 1994; Chen, 2002). HiPSC-CM lose their rod-shaped appearance during digestion very quickly. Freshly isolated hiPSC-CM do not adhere to the bottom of a recording chamber even if coated. We are sorry to report that we have failed to handle freshly isolated hiPSC-CM in electrophysiological experiments. Re-culturing hiPSC-CM from EHT in monolayer format could reverse some effects of EHT culture.

## Author contributions

AU, AHo, IM, and KB performed research. AU, AHo, AHa, TE, and TC planned experiments. AU, IM, KB, and SJ analyzed results. AU, TE, and TC wrote the manuscript. All authors approved the final version of the manuscript.

## Funding

The work was supported by the Graduate School of Cardiovascular Research Centre, Hamburg, the German Centre for Cardiovascular Research (DZHK) and the German Ministry of Education and Research (BMBF), the German Research Foundation (DFG Es 88/12-1) and the European Research Council (ERC AG IndivuHeart).

### Conflict of interest statement

We hereby confirm that any and all potential conflicts of interest have been fully and properly disclosed in the manuscript as outlined. IM, AH, and TE are co-founder of EHT Technologies GmbH, Hamburg. The other authors declare that the research was conducted in the absence of any commercial or financial relationships that could be construed as a potential conflict of interest.

## Abbreviations

5-HT: 5-hydroxytryptamine
AR: adrenoceptor
CM: cardiomyocytes
EHT: engineered heart tissue
Epi: epinephrine
HiPSC-CM: human induced pluripotent stem cell-derived cardiomyocytes
I_Ca,L_: L-type Ca^2+^- current
I_Ca,T_: T-type Ca^2+^-current
NE: norepinephrine
ML: monolayer
PKA: protein kinase A
TTX: tetrodotoxin.

## References

[B1] Beuckelmann D. J. Näbauer M. Erdmann E. (1991). Characteristics of calcium-current in isolated human ventricular myocytes from patients with terminal heart failure. J. Mol. Cell. Cardiol. 23, 929–937. 10.1016/0022-2828(91)90135-9 1658345

[B2] Bosch R. F. Zeng X. Grammer J. B. Popovic K. Mewis C. Kühlkamp V. (1999). Ionic mechanisms of electrical remodeling in human atrial fibrillation. Cardiovasc. Res. 44, 121–131. 10.1016/S0008-6363(99)00178-9 10615396

[B3] Brattelid T. Qvigstad E. Moltzau L. R. Bekkevold SVS, Sandnes, D. L. Birkeland J. A. K.. (2012). The cardiac ventricular 5-HT4 receptor is functional in late foetal development and is reactivated in heart failure. PLoS ONE 7:e45489. 10.1371/journal.pone.0045489 23029047 PMC3447799

[B4] Burridge P. W. Keller G. Gold J. D. Wu J. C. (2012). Production of *de novo* cardiomyocytes: human pluripotent stem cell differentiation and direct reprogramming. Cell Stem Cell 10, 16–28. 10.1016/j.stem.2011.12.013 22226352 PMC3255078

[B5] Burridge P. W. Matsa E. Shukla P. Lin Z. C. Churko J. M. Ebert A. D.. (2014). Chemically defined generation of human cardiomyocytes. Nat. Methods 11, 855–860. 10.1038/nmeth.2999 24930130 PMC4169698

[B6] Chen X. (2002). L-type Ca^2+^ channel density and regulation are altered in failing human ventricular myocytes and recover after support with mechanical assist devices. Circ. Res. 91, 517–524. 10.1161/01.RES.0000033988.13062.7C 12242270

[B7] Christ T. Boknik P. Wöhrl S. Wettwer E. Graf E. M. Bosch R. F.. (2004a). L-type Ca^2+^ current downregulation in chronic human atrial fibrillation is associated with increased activity of protein phosphatases. Circulation 110, 2651–2657. 10.1161/01.CIR.0000145659.80212.6A 15492323

[B8] Christ T. Molenaar P. Klenowski P. M. Ravens U. Kaumann A. J. (2011). Human atrial β1L-adrenoceptor but not β3-adrenoceptor activation increases force and Ca^2+^ current at physiological temperature. Br. J. Pharmacol. 162, 823–839. 10.1111/j.1476-5381.2010.00996.x 20726983 PMC3042194

[B9] Christ T. Rozmaritsa N. Engel A. Berk E. Knaut M. Metzner K.. (2014). Arrhythmias, elicited by catecholamines and serotonin, vanish in human chronic atrial fibrillation. Proc. Natl. Acad. Sci. U.S.A. 111, 11193–11198. 10.1073/pnas.1324132111 25024212 PMC4121801

[B10] Christ T. Schindelhauer S. Wettwer E. Wallukat G. Ravens U. (2006). Interaction between autoantibodies against the beta1-adrenoceptor and isoprenaline in enhancing L-type Ca^2+^ current in rat ventricular myocytes. J. Mol. Cell. Cardiol. 41, 716–723. 10.1016/j.yjmcc.2006.06.011 16889792

[B11] Christ T. Wettwer E. Dobrev D. Adolph E. Knaut M. Wallukat G.. (2001). Autoantibodies against the beta1 adrenoceptor from patients with dilated cardiomyopathy prolong action potential duration and enhance contractility in isolated cardiomyocytes. J. Mol. Cell. Cardiol. 33, 1515–1525. 10.1006/jmcc.2001.1414 11448139

[B12] Christ T. Wüst M. Matthes J. Jänchen M. Jürgens S. Herzig S.. (2004b). An aqueous extract of the marine sponge Ectyoplasia ferox stimulates L-type Ca^2+^-current by direct interaction with the Cav1.2 subunit. Naunyn Schmiedebergs. Arch. Pharmacol. 370, 474–483. 10.1007/s00210-004-0996-4 15599709

[B13] Dick E. Rajamohan D. Ronksley J. Denning C. (2010). Evaluating the utility of cardiomyocytes from human pluripotent stem cells for drug screening. Biochem. Soc. Trans. 38, 1037–1045. 10.1042/BST0381037 20659000

[B14] Dobrev D. Wettwer E. Himmel H. M. Kortner A. Kuhlisch E. Schüler S.. (2000). G-Protein beta(3)-subunit 825T allele is associated with enhanced human atrial inward rectifier potassium currents. Circulation 102, 692–697. 10.1161/01.CIR.102.6.692 10931811

[B15] Frank S. Zhang M. Schöler H. R. Greber B. (2012). Small molecule-assisted, line-independent maintenance of human pluripotent stem cells in defined conditions. PLoS ONE 7:e41958. 10.1371/journal.pone.0041958 22860038 PMC3408405

[B16] Hagiwara N. Irisawa H. Kameyama M. (1988). Contribution of two types of calcium currents to the pacemaker potentials of rabbit sino-atrial node cells. J. Physiol. 395, 233–253. 10.1113/jphysiol.1988.sp016916 2457676 PMC1191991

[B17] Hansen J. Chen R. Larsen J. Chu P. Janes D. Weis K.. (2004). Calcium channel γ6 subunits are unique modulators of low voltage-activated (Cav3.1) calcium current. J. Mol. Cell. Cardiol. 37, 1147–1158. 10.1016/j.yjmcc.2004.08.005 15572045

[B18] Hegyi B. Bárándi L. Komáromi I. Papp F. Horváth B. Magyar J.. (2012). Tetrodotoxin blocks L-type Ca^2+^ channels in canine ventricular cardiomyocytes. Pflügers Arch. Eur. J. Physiol. 464, 167–174. 10.1007/s00424-012-1114-y 22615072

[B19] Heubach J. F. Köhler A. Wettwer E. Ravens U. (2000). T-Type and tetrodotoxin-sensitive Ca^(2+)^ currents coexist in guinea pig ventricular myocytes and are both blocked by mibefradil. Circ. Res. 86, 628–635. 10.1161/01.RES.86.6.628 10746997

[B20] Hirano Y. Fozzard H. A. January C. T. (1989). Characteristics of L- and T-type Ca^2+^ currents in canine cardiac Purkinje cells. Am. J. Physiol. 256, H1478–H1492. 2470265 10.1152/ajpheart.1989.256.5.H1478

[B21] Hoekstra M. Mummery C. L. Wilde A. A. M. Bezzina C. R. Verkerk A. O. (2012). Induced pluripotent stem cell derived cardiomyocytes as models for cardiac arrhythmias. Front. Physiol. 3:346. 10.3389/fphys.2012.00346 23015789 PMC3449331

[B22] Hofmann F. Flockerzi V. Kahl S. Wegener J. W. (2014). L-type CaV1.2 calcium channels: from *in vitro* findings to *in vivo* function. Physiol. Rev. 94, 303–326. 10.1152/physrev.00016.2013 24382889

[B23] Jahnel U. Rupp J. Ertl R. Nawrath H. (1992). Positive inotropic response to 5-HT in human atrial but not in ventricular heart muscle. Naunyn Schmiedebergs. Arch. Pharmacol. 346, 482–485. 10.1007/BF00169000 1335123

[B24] Ji J. Kang J. Rampe D. (2014). L-type Ca^2+^ channel responses to Bay K 8644 in stem cell-derived cardiomyocytes are unusually dependent on holding potential and charge carrier. Assay Drug Dev. Technol. 12, 352–360. 10.1089/adt.2014.596 25147907 PMC4142808

[B25] Kaumann A. J. Hall J. A. Murray K. J. Wells F. C. Brown M. J. (1989). A comparison of the effects of adrenaline and noradrenaline on human heart: the role of beta 1- and beta 2-adrenoceptors in the stimulation of adenylate cyclase and contractile force. Eur. Heart J. 10(Suppl. B), 29–37. 2572419 10.1093/eurheartj/10.suppl_b.29

[B26] Kaumann A. J. Sanders L. Brown A. M. Murray K. J. Brown M. J. (1990). A 5-hydroxytryptamine receptor in human atrium. Br. J. Pharmacol. 100, 879–885. 10.1111/j.1476-5381.1990.tb14108.x 2169944 PMC1917575

[B27] Kawano S. DeHaan R. L. (1989). Low-threshold current is major calcium current in chick ventricle cells. Am. J. Physiol. 256, H1505–H1508. 2719143 10.1152/ajpheart.1989.256.5.H1505

[B28] Kawano S. DeHaan R. L. (1990). Analysis of the T-type calcium channel in embryonic chick ventricular myocytes. J. Membr. Biol. 116, 9–17. 10.1007/BF01871667 2165178

[B29] Lanier M. Schade D. Willems E. Tsuda M. Spiering S. Kalisiak J.. (2012). Wnt inhibition correlates with human embryonic stem cell cardiomyogenesis: a structure-activity relationship study based on inhibitors for the Wnt response. J. Med. Chem. 55, 697–708. 10.1021/jm2010223 22191557 PMC3335202

[B30] Lemaire S. Piot C. Seguin J. Nargeot J. Richard S. (1995). Tetrodotoxin-sensitive Ca^2+^ and Ba^2+^ currents in human atrial cells. Recept. Channels 3, 71–81. 8581402

[B31] Li G. R. Nattel S. (1997). Properties of human atrial ICa at physiological temperatures and relevance to action potential. Am. J. Physiol. 272, H227–H235. 9038942 10.1152/ajpheart.1997.272.1.H227

[B32] Ma J. Guo L. Fiene S. J. Anson B. D. Thomson J. A. Kamp T. J.. (2011). High purity human-induced pluripotent stem cell-derived cardiomyocytes: electrophysiological properties of action potentials and ionic currents. AJP Hear. Circ. Physiol. 301, H2006–H2017. 10.1152/ajpheart.00694.2011 21890694 PMC4116414

[B33] Marger L. Mesirca P. Alig J. Torrente A. Dübel S. Engeland B.. (2011). Functional roles of Ca v 1.3, Ca v 3.1 and HCN channels in automaticity of mouse atrioventricular cells. Channels 5, 251–261. 10.4161/chan.5.3.15266 21406960 PMC3225754

[B34] Maylie J. Morad M. (1995). Evaluation of T- and L-type Ca^2+^ currents in shark ventricular myocytes. Am. J. Physiol. 269, H1695–H1703. 7503267 10.1152/ajpheart.1995.269.5.H1695

[B35] Mesirca P. Torrente A. G. Mangoni M. E. (2014). T-type channels in the sino-atrial and atrioventricular pacemaker mechanism. Pflugers Arch. Eur. J. Physiol. 466, 791–799. 10.1007/s00424-014-1482-6 24573175

[B36] Mewes T. Ravens U. (1994). L-type calcium currents of human myocytes from ventricle of non-failing and failing hearts and from atrium. J. Mol. Cell. Cardiol. 26, 1307–1320. 10.1006/jmcc.1994.1149 7869391

[B37] Mitra R. Morad M. (1986). Two types of calcium channels in guinea pig ventricular myocytes. Proc. Natl. Acad. Sci. U.S.A. 83, 5340–5344. 10.1073/pnas.83.14.5340 2425366 PMC323947

[B38] Navarrete E. G. Liang P. Lan F. Sanchez-Freire V. Simmons C. Gong T. (2013). Screening drug-induced arrhythmia events using human induced pluripotent stem cell-derived cardiomyocytes and low-impedance microelectrode arrays. Circulation 128, S3–13. 10.1161/CIRCULATIONAHA.112.000570 24030418 PMC3855862

[B39] Nemtsas P. Wettwer E. Christ T. Weidinger G. Ravens U. (2010). Adult zebrafish heart as a model for human heart? An electrophysiological study. J. Mol. Cell. Cardiol. 48, 161–171. 10.1016/j.yjmcc.2009.08.034 19747484

[B40] Ono K. Iijima T. (2010). Cardiac T-type Ca^2+^ channels in the heart. J. Mol. Cell. Cardiol. 48, 65–70. 10.1016/j.yjmcc.2009.08.021 19729018

[B41] Ouadid H. Seguin J. Dumuis A. Bockaert J. Nargeot J. (1992). Serotonin increases calcium current in human atrial myocytes via the newly described 5-hydroxytryptamine receptors. Mol. Pharmacol. 41, 346–351. 1311410

[B42] Ouadid H. Séguin J. Richard S. Chaptal P. A. Nargeot J. (1991). Properties and Modulation of Ca channels in adult human atrial cells. J. Mol. Cell. Cardiol. 23, 41–54. 10.1016/0022-2828(91)90037-M 1709972

[B43] Qu Y. Boutjdir M. (2001). Gene expression of SERCA2a and L- and T-Type Ca channels during human heart development. Pediatr. Res. 50, 569–574. 10.1203/00006450-200111000-00006 11641449

[B44] R Core Team (2013). R: A Language and Environment for Statistical Computing. Vienna: R Foundation for Statistical Computing.

[B45] Ravens U. Schoepper H-P. (1990). Opposite cardiac actions of the enantiomers of Bay K 8644 at different membrane potentials in guinea-pig papillary muscles. Naunyn Schmiedebergs. Arch. Pharmacol. 341, 232–239. 10.1007/BF00169736 1692975

[B46] Reuter H. (1974). Localization of beta adrenergic receptors, and effects of noradrenaline and cyclic nucleotides on action potentials, ionic currents and tension in mammalian cardiac muscle. J. Physiol. 242, 429–451. 10.1113/jphysiol.1974.sp010716 4376168 PMC1330676

[B47] Schaaf S. Eder A. Vollert I. Stöhr A. Hansen A. Eschenhagen T. (2014). Generation of strip-format fibrin-based engineered heart tissue (EHT), in Card Tissue Engineering Methods Molecular Biology, Vol. 1181, eds Radisic M. Black L. D III (New York, NY: Springer); 121–129. 10.1007/978-1-4939-1047-2_11 25070332

[B48] Schaaf S. Shibamiya A. Mewe M. Eder A. Stöhr A. Hirt M. N.. (2011). Human engineered heart tissue as a versatile tool in basic research and preclinical toxicology. PLoS ONE 6:e26397. 10.1371/journal.pone.0026397 22028871 PMC3197640

[B49] Schoemaker R. Du X. Bax W. Saxena P. (1992). 5-Hydroxytryptamine increases contractile force in porcine right atrium but not in left ventricle. Naunyn Schmiedebergs. Arch. Pharmacol. 346, 486–489. 10.1007/BF00169001 1470219

[B50] Schramm M. Thomas G. Towart R. Franckowiak G. (1983). Novel dihydropyridines with positive inotropic action through activation of Ca^2+^ channels. Nature 303, 535–537. 10.1038/303535a0 6190088

[B51] Tseng G. N. Boyden P. A. (1991). Different effects of intracellular Ca and protein kinase C on cardiac T and L Ca currents. Am. J. Physiol. 261, H364–H379. 1652211 10.1152/ajpheart.1991.261.2.H364

[B52] Tsien R. W. (1983). Calcium channels in excitable cell membranes. Annu. Rev. Physiol. 45, 341–358. 10.1146/annurev.ph.45.030183.002013 6303205

[B53] Van Haastert P. J. M. Van Driel R. Jastorff B. (1984). Competitive cAMP antagonists for cAMP-receptor proteins. J. Biol. Chem. 259, 10020–10024. 6088478

[B54] Wilson J. R. Clark R. B. Banderali U. Giles W. R. (2011). Measurement of the membrane potential in small cells using patch clamp methods. Channels 5, 530–537. 10.4161/chan.5.6.17484 21829090 PMC3265801

[B55] Xu X. Best P. M. (1992). Postnatal changes in T-type calcium current density in rat atrial myocytes. J. Physiol. 454, 657–672. 10.1113/jphysiol.1992.sp019285 1335509 PMC1175626

[B56] Yang X. Pabon L. Murry C. E. (2014). Engineering adolescence: maturation of human pluripotent stem cell-derived cardiomyocytes. Circ. Res. 114, 511–523. 10.1161/CIRCRESAHA.114.300558 24481842 PMC3955370

[B57] Yazawa M. Hsueh B. Jia X. Pasca A. M. Bernstein J. A. Hallmayer J.. (2011). Using induced pluripotent stem cells to investigate cardiac phenotypes in Timothy syndrome. Nature 471, 230–234. 10.1038/nature09855 21307850 PMC3077925

[B58] Zweigerdt R. Olmer R. Singh H. Haverich A. Martin U. (2011). Scalable expansion of human pluripotent stem cells in suspension culture. Nat. Protoc. 6, 689–700. 10.1038/nprot.2011.318 21527925

