# Supplementary material for: Ca2+-Currents in Human Induced Pluripotent Stem Cell-Derived Cardiomyocytes Effects of Two Different Culture Conditions
Source: Front Pharmacol. 2016 Sep 12;7:300. doi: 10.3389/fphar.2016.00300 (PMC5018497; doi:10.3389/fphar.2016.00300)
Supplement: Supplementary file 1 [file DataSheet1.DOCX]

**Supplementary material**

**Figure S1: T-type and L-type Ca^2+^-current are insensitive to TTX**

**A, B**:Time course of I_Ca,L_ and I_Ca,T_ in EHT exposed to TTX (30 µM). Original tracings taken at time points indicated as **a** and **b.** Respective pulse protocol given as inset on top. Please note that both I_Ca,T_ and I_Ca,L_ were not blocked. **C**: Mean values for I_Ca,T_ and I_Ca,L_ before and after exposure to TTX (30 µM). N/N indicates number of cells vs. number of isolation for EHT.

**Figure S2: Positive correlation between effect size and basal current density**

Individual data points and respective mean values for cells successfully exposed to catecholamines. Dotted lines indicate linear regression. N/N indicates number of cells vs. number of isolation for ML and EHT and number of cells/number patients for atrial and ventricular CM.

**Figure S3: T-type Ca^2+^-current is insensitive to β-AR-stimulation**

**A, B**: Time course of I_Ca,L_ and I_Ca,T_ in EHT exposed to norepinephrine (NE, 100 µM). Original tracings taken at time points indicated as **a** and **b.** Respective pulse protocol given as inset on top. Please note that I_Ca,T_ remained constant upon β-AR-stimulation while I_Ca,L_ is increased. **C**: Mean values for I_Ca,T_ and I_Ca,L_ before and after exposure to norepinephrine (NE, 100 µM). N/N indicates number of cells vs. number of isolation for EHT.

**Figure S4: Temperature-dependency of catecholamine-induced response**

**A, B**: Basal current densities as well as increases upon β_1_-AR (**A**) and β_2_-AR (**B**) stimulation in hiPSC-CM (EHT) in experiments at room temperature (RT) vs. 37 °C. **C**: Increases for norepinephrine (NE, 100 µM) given as absolute delta values (bars on left-hand side) and in percent of increase (bars on right-hand side). **D**: Increases for epinephrine (Epi, 100 µM). Same layout as in **C.** N/N indicates number of cells vs. number of isolation for EHT.

**Table 1: Patient characteristics**

**Atrial and ventricular CM from patients used for experiments.**

|  | **Atrial** | **Ventricular** | |  |
| --- | --- | --- | --- | --- |
| N | 6 | | 14 |  |
| Gender [m/f] | 4 / 2 | | 9 / 5 |  |
| Age [years] | 61.7 ± 2.7 | | 61.6 ± 3.9 |  |
| BMI [kg/m^2^] | 28.7 ± 2.6 | | 27.1 ± 0.7 |  |
| Hypertension, n | 5 | | 7 |  |
| Diabetes mellitus, n | 3 | | 5 |  |
| Hyperlipidaemia, n | 5 | | 5 |  |
| CAD, n | 4 | | 4 |  |
| VD | 2 | | 10 |  |
| LVEF [%] | 60.7 ± 4.0 | | 41.0 ± 4.7 |  |
|  |  | |  |  |
| LA [mm] | 40. ± 4.0 | | 49.3 ± 2.1 |  |
| LVEDD [mm] | 45.3 ± 1.9 | | 56.9 ± 4.8 |  |
| Cardiovascular medication (n) |  | |  |  |
| Digitalis | 2 | | 2 |  |
| ACE-Inhibitors | 4 | | 5 |  |
| AT_1_-blockers | 0 | | 2 |  |
| β-blockers | 4 | | 5 |  |
| Ca^2+^-channel-blockers | 3 | | 0 |  |
| Diuretics | 0 | | 7 |  |
| Nitrates, n | 1 | | 0 |  |
| Lipid-lowering drugs | 4 | | 6 |  |

Abbreviations: AT, angiotensin receptor; VD, valve disease; CAD, coronary artery disease; LA, left atrial diameter; LVEDD, left ventricular end-diastolic diameter; LVEF, left ventricular ejection fraction; MVD, mitral valve disease

| **Table 2:** **Capacitance, descriptive statistics (for Fig. 1A).** | | | | | | | | | | |
| --- | --- | --- | --- | --- | --- | --- | --- | --- | --- | --- |
|  | **n** | **mean** | **median** | **min** | **max** | **skew** | **kurtosis** | **se** |  |  |
| **ML** | 289 | 45.3 | 40 | 6 | 131 | 0,9 | 0.5 | 1.4 |  |  |
| **EHT** | 460 | 45.2 | 41.5 | 8 | 132 | 0.9 | 0.8 | 1.1 |  |  |
| **Atrial** | 196 | 86.7 | 89 | 13 | 166 | -0,1 | -0.4 | 2.8 |  |  |
| **Ventricular** | 50 | 125.7 | 116 | 44 | 341 | 1.3 | 2.6 | 8.0 |  |  |

| **Table 3: Fit values for correlation between cell size and I_Ca_ amplitude shown in Fig. 1B.**   \|  \| **ML** \| **EHT** \| **Atrial** \| **Ventricular** \| \| --- \| --- \| --- \| --- \| --- \| \| **Slope** \| 11.1 ± 1.4^*,#^ \| 16.3 ± 1.1^*,#^ \| 4.6 ± 0.7 \| 4.6 ± 1.2 \| \| **Goodness of fit:** \|  \|  \|  \|  \| \| **R square** \| 0.2812 \| 0.3998 \| 0.1848 \| 0.2339 \| \| **Is slope significantly non-zero?** \|  \|  \|  \|  \| \| **P value** \| < 0.0001 \| < 0.0001 \| < 0.0001 \| 0,0006 \| \| **Deviation from zero?** \| Significant \| Significant \| Significant \| Significant \| \|  \|  \|  \|  \|  \| \|  \|  \|  \|  \|  \| |
| --- | --- | --- | --- | --- | --- | --- | --- | --- | --- | --- | --- | --- | --- | --- | --- | --- | --- | --- | --- | --- | --- | --- | --- | --- | --- | --- | --- | --- | --- | --- | --- | --- | --- | --- | --- | --- | --- | --- | --- | --- | --- | --- | --- | --- | --- |
| *significantly different from atrial, ^#^ significantly different from ventricular. |
| \| **Table 4: Current density, descriptive statistics (for Fig. 1C).** \| \| \| \| \| \| \| \| \| \| \| \| --- \| --- \| --- \| --- \| --- \| --- \| --- \| --- \| --- \| --- \| --- \| \|  \| **n** \| **mean** \| **median** \| **min** \| **max** \| **skew** \| **kurtosis** \| **se** \|  \|  \| \| **ML** \| 207 \| 9.9 \| 8.4 \| 0.4 \| 131.2 \| 7 \| 63.2 \| 0.9 \|  \|  \| \| **EHT** \| 361 \| 12.2 \| 10.2 \| 0.4 \| 85.9 \| 2.8 \| 14.1 \| 0.5 \|  \|  \| \| **Atrial** \| 196 \| 7.1 \| 6.3 \| 1.7 \| 22.4 \| 1.4 \| 1.9 \| 0.3 \|  \|  \| \| **Ventricular** \| 47 \| 5.7 \| 4.9 \| 1.3 \| 15.4 \| 1.2 \| 1.0 \| 0.5 \|  \|  \| |

**Table 5: Voltage dependency of current** **activation, steady state inactivation.**

| **Voltage-dependent inactivation** | **ML** | **EHT** | |
| --- | --- | --- | --- |
|  |  |  | |
| V_0.5_ (mV), I_Ca,T_ | -68.5±1.0 (12/6) | | -72.3±2.1 (7/5) |
| k_0.5_ | -6.2±0.4 | -5.1±1.4 | |
|  |  |  | |
|  |  |  | |
| V_0.5_ (mV), I_Ca,L_ | -31.3±1.7 (12/6) | -32.6±0.6 (7/5) | |
| k_0.5_ | -4.8±0.5 | -4.1±1.1 | |
|  |  |  | |
|  |  |  | |
| **Voltage-dependent activation** |  |  | |
|  |  |  | |
| Amplitude_ICa,T_ (%) | 11.5±0.5 (29/16) | 13±2.1(42/24) | |
| V_0.5,ICa,T_ (mV) | -32.3±3.1 | -35.1±2.6 | |
| Slope_ICa,T_ | 3.9±0.4 | 4.8±0.8 | |
| V_0.5,ICa,L_ (mV) | -8.2±1.0 | -6.0±0.9 | |
| Slope_ICa,L_ | 5.2±0.4 | 4.9±0.2 | |
|  |  |  | |
|  |  |  | |
|  |  |  | |
| **Time-dependent inactivation** |  |  | |
| τ_slow_ (at +10 mV) | 25.4±3.1 (6/5) | 22.4±2.1 (9/6) | |
| τ_fast_ (at +10 mV) | 5.0±0.7 | 3.8±0.3 | |
| A (at +10 mV) | -333.7±64.9 | -269.4±23.6 | |
| B (at +10 mV) | -207.3±58.6 | -245.7±47.3 | |
|  |  |  | |
|  |  |  | |
|  |  |  | |
|  |  |  | |

|  |  |  |  |
| --- | --- | --- | --- |
|  |  |  |  |
|  |  |  |  |
|  |  |  |  |
|  |  |  |  |
|  |  |  |  |
