# Supplementary figures and images for: Ca2+-Currents in Human Induced Pluripotent Stem Cell-Derived Cardiomyocytes Effects of Two Different Culture Conditions
Source: Front Pharmacol. 2016 Sep 12;7:300. doi: 10.3389/fphar.2016.00300 (PMC5018497; doi:10.3389/fphar.2016.00300)

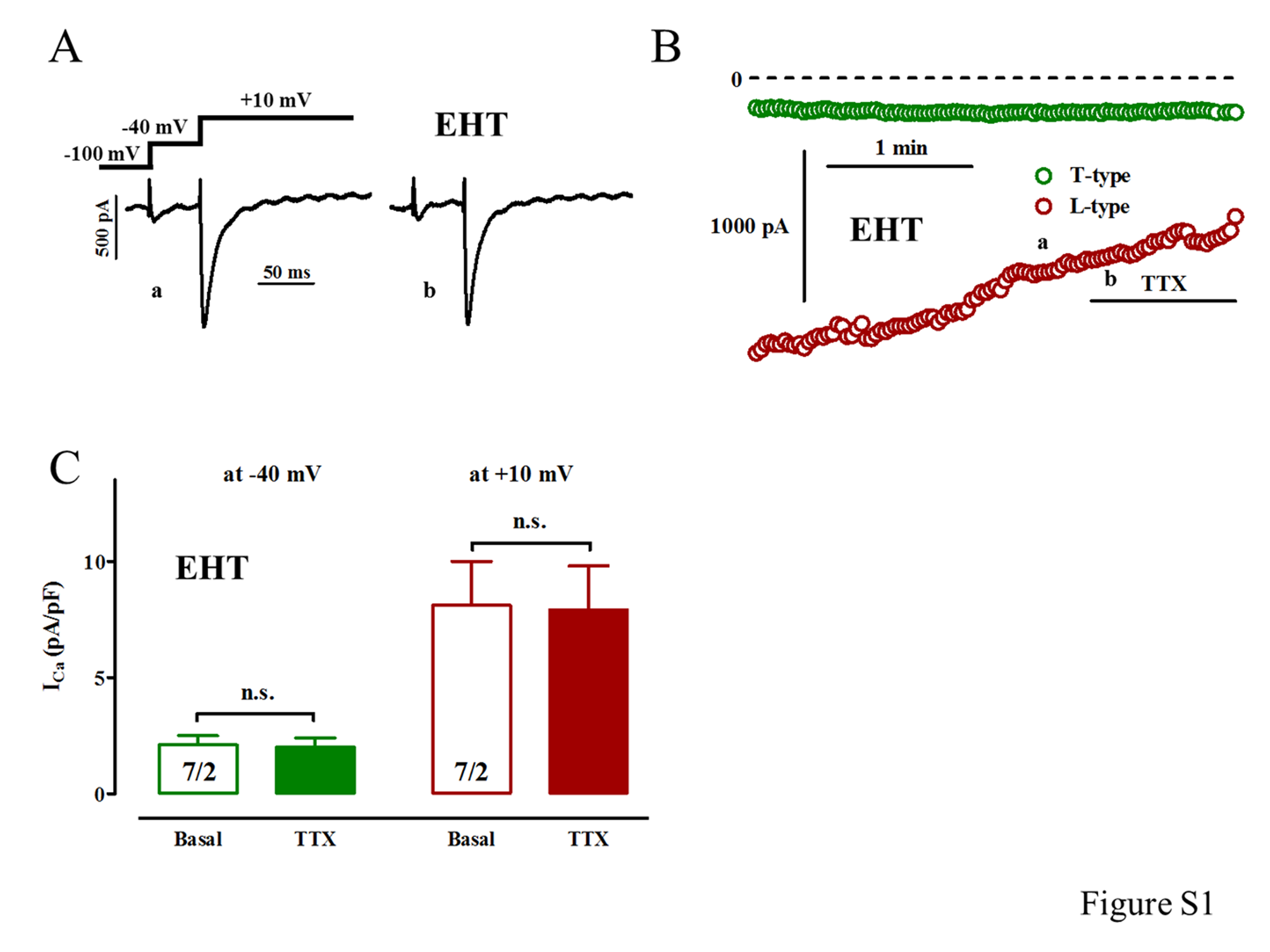

Supplement: Supplementary file 2 [file Image1.TIF]

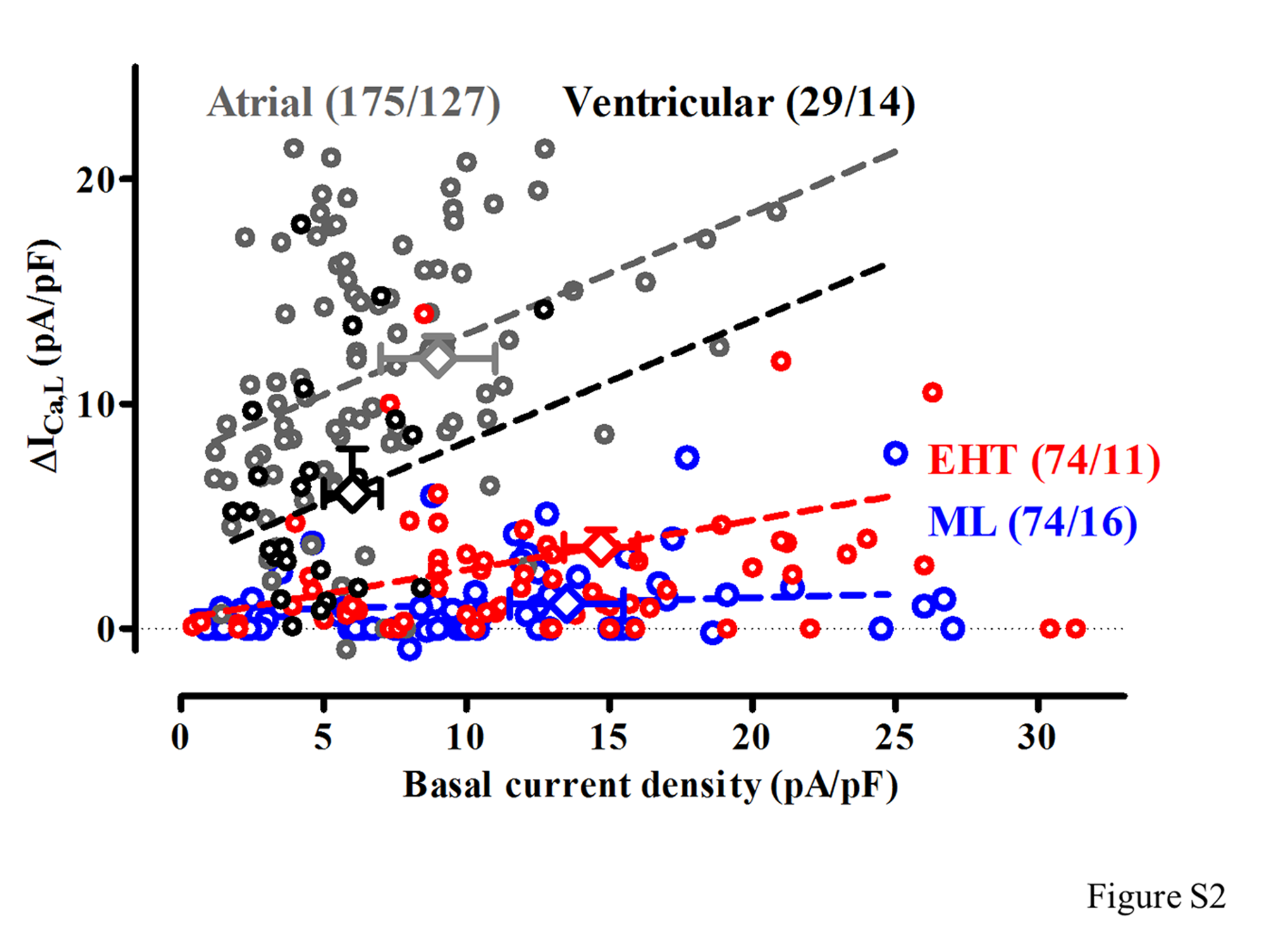

Supplement: Supplementary file 3 [file Image2.TIF]

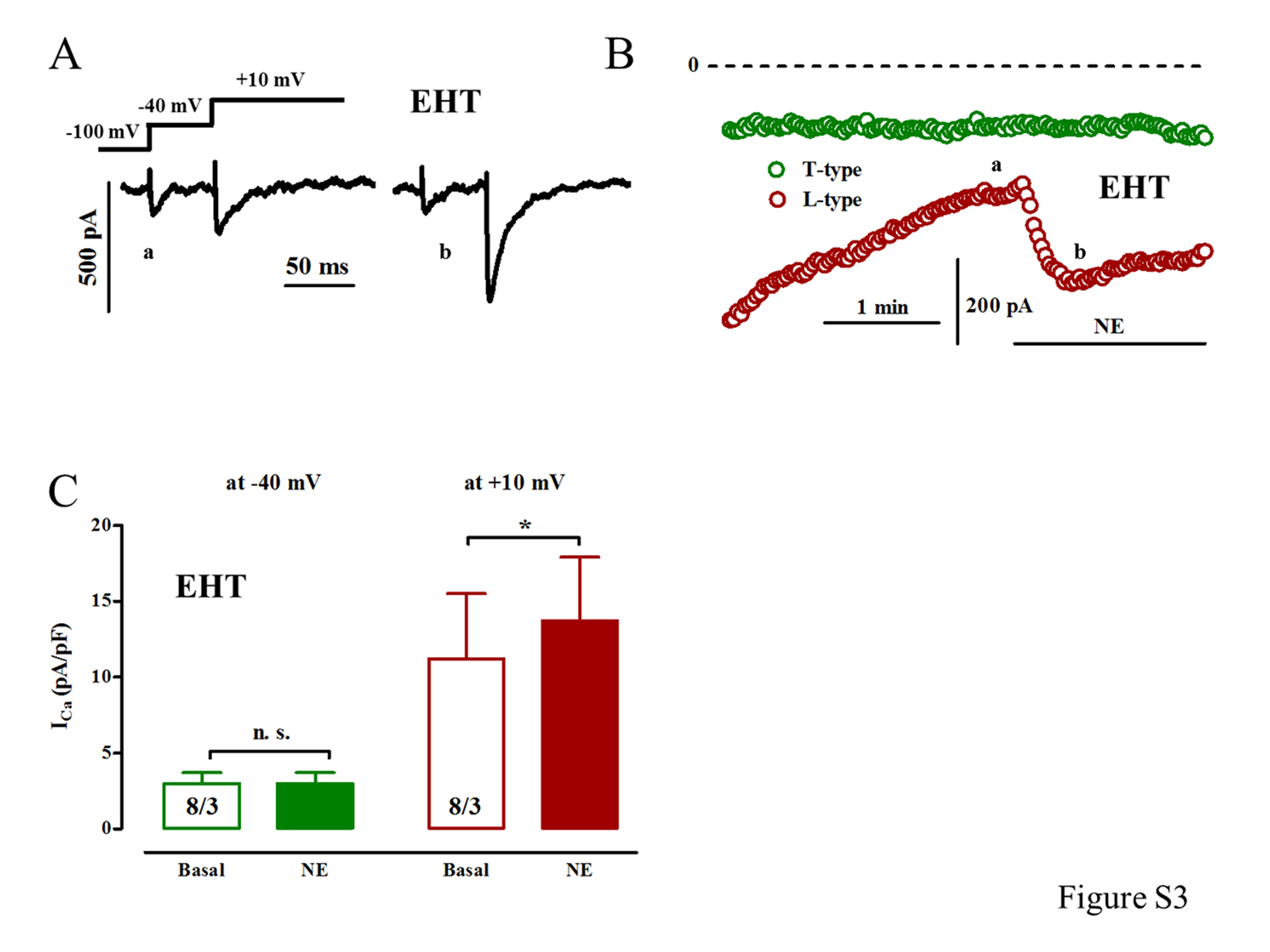

Supplement: Supplementary file 4 [file Image3.TIF]

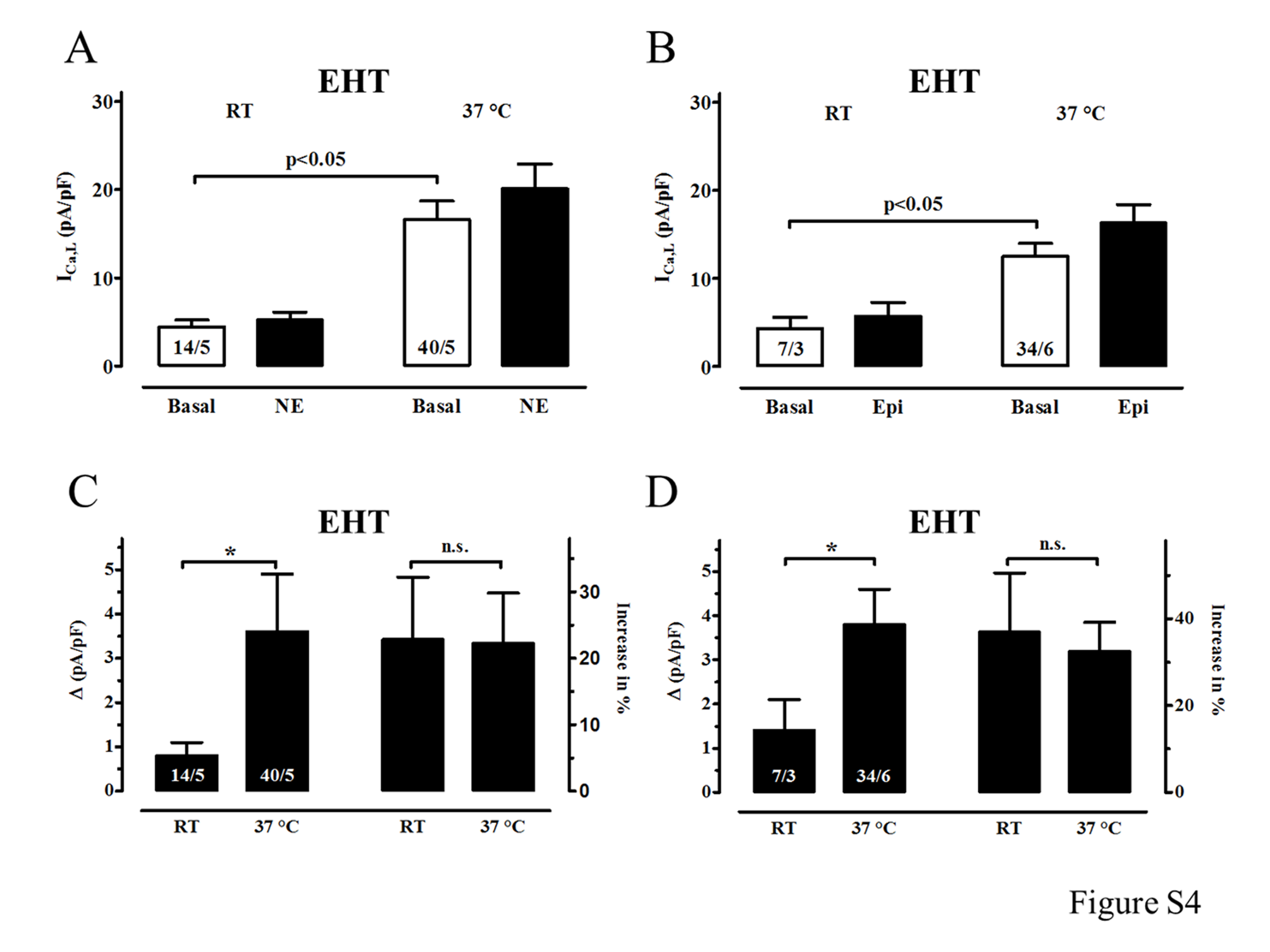

Supplement: Supplementary file 5 [file Image4.TIF]
